# Learned behavioral avoidance can alter outbreak dynamics in a model for waterborne infectious diseases

**DOI:** 10.1007/s00285-025-02252-7

**Published:** 2025-08-18

**Authors:** Anna J. Poulton, Stephen P. Ellner

**Affiliations:** 1 Center for Applied Mathematics, Cornell University, Ithaca, NY 14853 USA; 2 Department of Ecology and Evolutionary Biology, Cornell University, Ithaca, NY 14853 USA; 3 Present Address: Institute of Marine Sciences, Fisheries Collaborative Program, University of California, Santa Cruz, CA 95060 USA

**Keywords:** Avoidance behavior, Backward bifurcation, Chytridiomycosis, Environmental transmission, Infectious diseases, Stability, 92D30, 37N25, 34C60, 92D40

## Abstract

Many animals show avoidance behavior in response to disease. For instance, in some species of frogs, individuals that survive infection of the fungal disease chytridiomycosis may learn to avoid areas where the pathogen is present. As chytridiomycosis has caused substantial declines in many amphibian populations worldwide, it is a highly relevant example for studying these behavioral dynamics. Here we develop compartmental ODE models to study the epidemiological consequences of avoidance behavior of animals in response to waterborne infectious diseases. Individuals with avoidance behavior are less likely to become infected, but avoidance may also entail increased risk of mortality. We compare the outbreak dynamics with avoidance behavior that is innate (present from birth) or learned (gained after surviving infection). We also consider how management to induce learned avoidance might affect the resulting dynamics. Using methods from dynamical systems theory, we calculate the basic reproduction number $R_0$ for each model, analyze equilibrium stability of the systems, and perform a detailed bifurcation analysis. We show that disease persistence when $R_0< 1$ is possible with learned avoidance, but not with innate avoidance. Our results imply that management to induce behavioral avoidance can actually cause such a scenario, but it is also less likely to occur for high-mortality diseases (e.g., chytridiomycosis). Furthermore, the learned avoidance model demonstrates a variety of codimension-1 and -2 bifurcations not found in the innate avoidance model. Simulations with parameters based on chytridiomycosis are used to demonstrate these features and compare the outcomes with innate, learned, and no avoidance behavior.

**Supplementary Information:**

The online version contains supplementary material available at 10.1007/s00285-025-02252-7.

## Introduction

Resistance mechanisms are broadly defined as ways in which infection can be prevented or reduced (Miller et al. 2005; Roy and Kirchner 2000). The immune response is an important such mechanism, but other paths to resistance may affect host-pathogen dynamics in different ways (Reluga and Medlock 2007). Here we focus on avoidance behavior, through which individuals attempt to escape becoming infected by avoiding environments or interactions that carry an infection risk (Behringer et al. 2018). Examples from across a variety of taxa include avoiding interactions with infected conspecifics (mice: Boillat et al. 2015; frogs: Herczeg et al. 2024), avoiding consuming potentially contaminated food or water (elephants: Ndlovu et al. 2018; bonobos: Sarabian et al. 2018; nematodes: Zhang et al. 2005), and avoiding the pathogen (or some indicator of its presence) in the environment directly (frogs: McMahon et al. 2021, termites: Rosengaus et al. 1999). The level of avoidance expressed by an individual at a specific time may or may not depend on disease prevalence. For instance, animals may express avoidance by limiting contact with all conspecifics, or may only avoid interacting with conspecifics that appear to be infected, in which case the level of behavioral expression is dependent on disease prevalence in the population (Herczeg et al. 2024). Additionally, while avoidance behavior can reduce the chance of becoming infected, it may also carry fitness trade-offs: for example, if it decreases opportunities to obtain food or increases predation risk (Behringer et al. 2018). Although our focus here is on infectious diseases, very similar concepts of avoidance behavior also apply to parasitism (Ezenwa et al. 2022).

We broadly classify avoidance behaviors in response to disease into two categories: innate avoidance and learned avoidance. Innate avoidance behavior is present in individuals without any prior exposure or experience with the pathogen/disease. For instance, juvenile agile frogs (*R. dalmatina*) tended to avoid conspecifics infected with Ranavirus, even with no prior exposure to the virus (Herczeg et al. 2024). Mice have similarly been shown to express innate avoidance of infected conspecifics (Boillat et al. 2015). On the other hand, learned avoidance behavior is only acquired after experience with the pathogen (e.g., after recovering from an infection). For instance, both fruit flies (*D. melanogaster*) and nematodes (*C. elegans*) have been shown to express learned avoidance of pathogenic bacteria (Babin et al. 2014; Zhang et al. 2005). It is not always clear from observation alone whether avoidance behavior is innate or learned, but behavioral experiments can be used to distinguish between the two (e.g., McMahon et al. 2021).

An especially interesting example of learned avoidance behavior occurs in response to chytridiomycosis, a disease caused by the Bd (*Batrachochytrium dendrobatidis*) fungus that affects many amphibian species worldwide (Scheele et al. 2019). The Bd fungus is spread by waterborne zoospores that are shed by infected hosts (Berger et al. 2005). Experiments by McMahon et al. (2014, 2021) showed that in some species of frogs, individuals that experienced a chytridiomycosis infection could learn to avoid areas where the Bd fungus was present. The authors suggest that for amphibian species with the capacity for learned avoidance, inducing this behavior in individuals could be a viable management strategy for protecting vulnerable populations of amphibians and increasing the success of reintroduction efforts. As chytridiomycosis often carries a high mortality risk and has caused declines or extinctions in hundreds of amphibian species (Rosenblum et al. 2010; Scheele et al. 2019), learned avoidance behavior and its implications for management deserve further research attention. Thus, we use chytridiomycosis as our primary case study in this paper. While transmission of Bd may also be possible through direct contact (Rowley and Alford 2007), we focus our attention on environmental transmission of disease (e.g., via water or even moist sand/soil (Johnson and Speare 2005) and vegetation (Kolby et al. 2015)).

While modeling behavioral avoidance has received little attention in wildlife host-pathogen systems, avoidance behavior has been included in many models of human disease dynamics (Verelst et al. 2016). Such models have considered how human behavior may affect the outcome of epidemics, and how it can interact with other factors such as information availability, economic indicators, disease prevention measures, and more (Verelst et al. 2016). For instance, (Dashtbali and Mirzaie 2021) modeled how people altering their social distancing practices in response to reported death rates affected the dynamics of COVID-19. Similarly, (Yang et al. 2017) examined the effects of awareness programs (which were assumed to increase avoidance) in a model of cholera dynamics. Other models in human behavioral epidemiology have considered the impacts of vaccination (e.g. Bhattacharyya and Bauch 2011), hygienic behavior (e.g. masking or hand washing, (Wang et al. 2014)), social networks (e.g. Ni et al. 2011), and more.

While our models share some features with human-centric infectious disease models, there are several key differences. For one, human disease models often consider constant population sizes, or that population dynamics are not greatly altered by infection (McCallum 2016). In contrast, examining disease-induced mortality and its impacts on population size and population dynamics is a key question for many diseases of wildlife, including our chytrid example (McCallum 2016). Furthermore, we include a survival cost of avoidance in our model (e.g., due to increased predation risk or decreased access to food-rich areas), which is unlikely to be included in a human-centric disease model. Finally, the goals and policies used for disease management may differ greatly between human and wildlife systems (McCallum 2016).

Here we develop two compartmental ordinary differential equation models to study the epidemiological consequences of innate and learned avoidance behavior in animals in response to waterborne (or more generally, environmentally-transmitted) infectious diseases. The level of behavioral avoidance, along with the associated survival cost, are represented generally and allowed to depend on the current pathogen concentration in the environment. Using methods from dynamical systems theory, we calculate important properties for each model such as the basic reproduction number $R_0$ and the existence/stability of equilibrium points, and carry out a detailed bifurcation analysis. Special attention is given to the impacts of management effort to induce behavior in the learned avoidance system. We show that learned avoidance can lead to complex dynamics, including a variety of codimension-1 and -2 bifurcations, that are not present in the model of innate avoidance. Additionally, we use simulations to compare the outcomes of learned vs. innate vs. no avoidance, with parameters based on chytridiomycosis in frogs. Our work shows that with respect to increasing host population size, the relative performance of behavior types depends strongly on the mortality regime and the effectiveness of behavior at avoiding disease.

## Model formulation

### Model equations

The forms of our behavioral avoidance models are inspired by the work of (Reluga and Medlock 2007), although we make several changes to focus on wildlife disease modeling (specifically chytrid). Notably, we: (1) include disease-induced mortality, (2) examine environmental transmission via a pathogen pool, (3) consider non-constant avoidance behavior, and (4) include a potential survival cost of avoidance behavior.

**Fig. 1:**
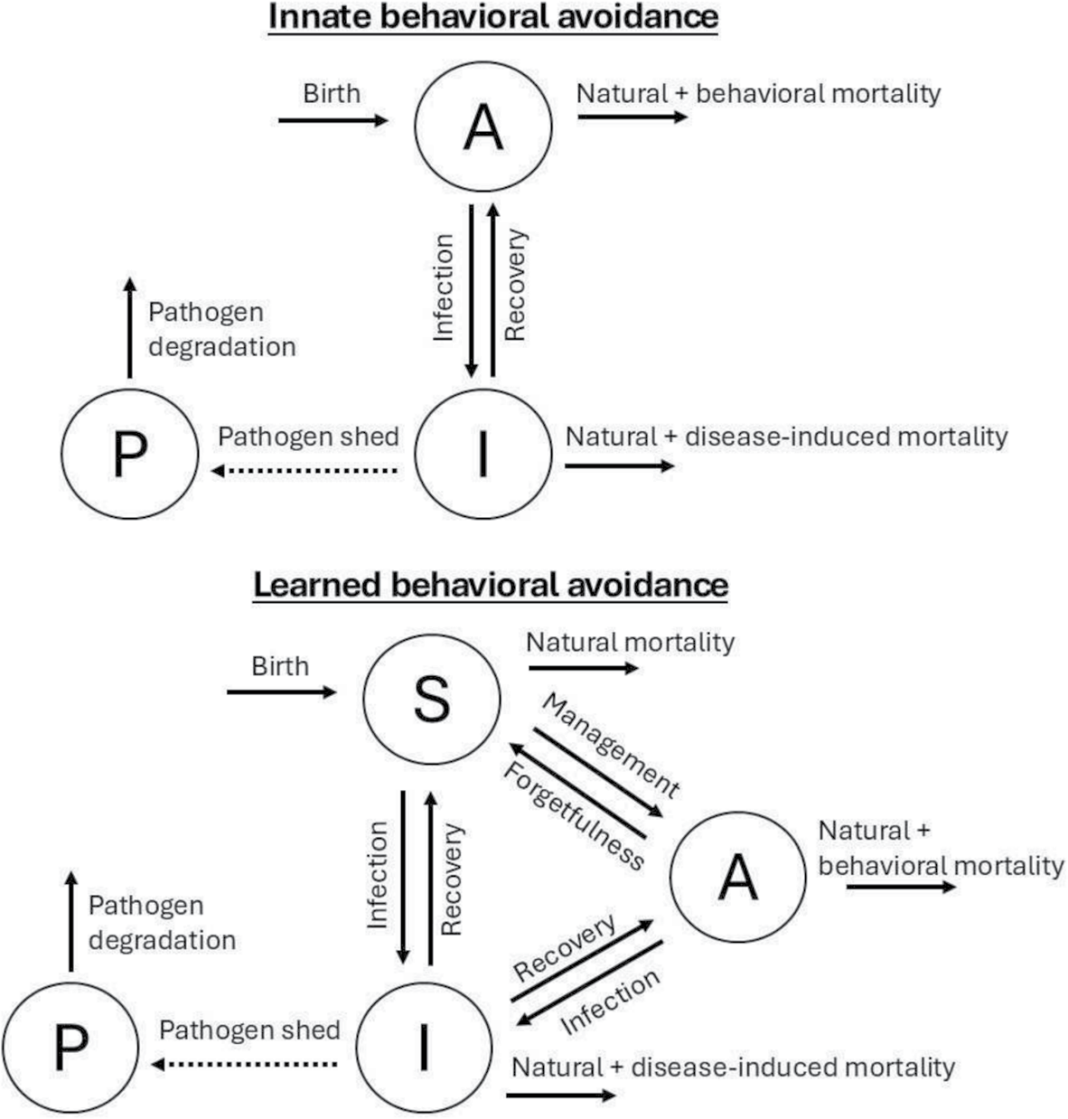
Diagrams summarizing the dynamics of the innate avoidance system (Eqs. 1-3; top) and the learned avoidance system (Eqs. 5-8; bottom). The compartments represent susceptible individuals without avoidance behavior (*S*), susceptible individuals with avoidance behavior (*A*), infected individuals (*I*), and the pathogen pool (*P*).

We consider two alternative models of behavioral avoidance, which are each summarized in Fig. 1. The first model we examine is the innate avoidance model (Eqs. 1-3), in which all individuals are born with avoidance behavior and the behavior cannot be lost. The compartments of this model represent susceptible individuals with avoidance behavior (A), infected individuals (I), and the pathogen pool (P). Infected individuals shed pathogens into the pool, which in turn infect susceptible individuals. Avoidance behavior is characterized in our model by two functions, $\alpha$ and *c*, which both potentially depend on the current pathogen pool concentration. The function $\alpha$ scales the base infection rate and represents the effectiveness of behavior at avoiding infection. It is unitless and constrained by $0 < \alpha (P) \le 1$, with $\alpha (P)$ near 1 representing weak avoidance and $\alpha (P) \ll 1$ representing strong avoidance. The function *c* represents a potential survival cost of avoidance behavior (e.g., if it leads to fewer opportunities to obtain food, or greater exposure to predation), and adds to the natural mortality rate for individuals with avoidance behavior. It is constrained by $c(P) \ge 0$, and has units of days$^{-1}$.

The innate avoidance system is given by:

1 $$\begin{aligned} \frac{dA}{dt} = \ &\mu -\beta \alpha (P) AP + \gamma I - (\mu + c (P)) A \, \end{aligned}$$

2 $$\begin{aligned} \frac{dI}{dt} = \ &\beta \alpha (P) AP - ( \gamma + \mu + \phi ) I \, \end{aligned}$$

3 $$\frac{{dP}}{{dt}} = \;\theta I - \lambda P.$$

The dynamics for the total population size ($N = A + I$) of the innate avoidance system are thus

4 $$\frac{{dN}}{{dt}} = \mu  - \mu N - c(P)A - \phi I.$$

We assume both $\alpha$ and *c* are $C^2$ (twice continuously differentiable) functions defined for $P \ge 0$, and furthermore that avoidance behavior should not decrease as pathogen concentration increases: $\alpha'(P) \le 0$ and $c'(P) \ge 0$. The simplest choices for $\alpha$ and *c* are constant functions, in which case we often use the notation $\alpha (P) = \alpha _0$ and $c(P) = c_0$. Note that if $\alpha _0 = 1$ and $c_0 = 0$, Eqs. 1-3 reduce to a model without behavior.

We assume that birth (or recruitment) into the population is constant, given by the parameter $\mu > 0$. Note that we also selected $\mu$ as the natural mortality rate because this results in the equilibrium population size being 1 when there are no other causes of mortality. We thus essentially treat our state variables as unitless, scaled quantities. The parameter $\beta >0$ represents the base infection rate for susceptible individuals (which is then modified by the avoidance function $\alpha$). Infected individuals may die due to natural mortality, die from disease-induced mortality ($\phi \ge 0$), or recover from infection at rate $\gamma > 0$. We further assume that $c(P) \le \phi$, such that mortality from behavioral avoidance is never greater than mortality from the disease itself. Finally, pathogens are released by infected individuals at the rate $\theta > 0$, and are lost over time at the rate $\lambda > 0$.

The second model we examine is the learned avoidance model (Eqs. 5 - 8). In this model, avoidance behavior is only gained after surviving an infection event, and may be forgotten over time. The compartments *A*, *I*, *P* are the same as described for the innate avoidance model, but now there is an additional compartment *S* representing fully susceptible individuals (no behavioral avoidance). Note that all individuals are born fully susceptible, unlike in the innate avoidance model.

5 $$\begin{aligned} \frac{dS}{dt} = \ &\mu - \beta S P + (1-p)\gamma I + wA - vS - \mu S \, \end{aligned}$$

6 $$\begin{aligned} \frac{dA}{dt} = \ &-\beta \alpha (P) AP + p\gamma I - w A + vS - (\mu + c(P)) A \, \end{aligned}$$

7 $$\begin{aligned} \frac{dI}{dt} = \ &\beta SP + \beta \alpha (P) AP - (\gamma + \mu + \phi ) I \, \end{aligned}$$

8 $$\frac{{dP}}{{dt}} = \;\theta I - \lambda P.$$

The dynamics for total population size of the learned avoidance system are the same as in the innate avoidance model, but with $N = S + A + I$:

9 $$\frac{{dN}}{{dt}} = \mu  - \mu N - c(P)A - \phi I.$$

There are two paths to learning avoidance behavior. The first is through surviving a natural infection event in the wild: among infected individuals *I* that recover, a proportion *p* move into *A* while $1-p$ move into *S*. The parameter *p* effectively represents the probability that avoidance behavior is learned post infection, with $0 \le p \le 1$. The second path to avoidance behavior is also through infection, but in a controlled setting as the result of management effort. For instance, concerning frogs and chytrid, heat treatments can potentially be used to clear frogs of infection safely while still inducing avoidance behavior (as in McMahon et al. 2014, 2021). The parameter $v \ge 0$ represents a constant rate of management effort put into the system to induce avoidance behavior, resulting in individuals going from $S \rightarrow A$ (as we assume for simplicity that this action carries no risk of disease-induced mortality). Finally, learned behavioral avoidance may be forgotten over time at rate $w \ge 0$. All other parameters are as described for the innate avoidance model.

Additionally, one important derived parameter is the basic reproduction number $R_0$, which represents the number of secondary cases caused by an infected individual in an otherwise susceptible population (Diekmann et al. 1990). We calculate this for each of our models using the next generation matrix approach described in (van den Driessche and Watmough 2002). Typically, we expect an infectious disease to die out when $R_0 < 1$ and persist if $R_0 > 1$. However, in certain scenarios disease persistence may be possible even when $R_0 < 1$ due to a ‘backward bifurcation’, which we describe more in Sect. 3.3.

### Parameter values

Much of our work is analytical and does not require selecting specific parameter values, but for numerical/simulation results when possible we selected parameter values using chytridiomycosis in frogs as our case study (Table 1). It should be noted that parameters for chytridiomycosis dynamics can vary over a large range due to differences between species and environmental factors (such as temperature) (Rosenblum et al. 2010; Tobler and Schmidt 2010; Wilber et al. 2016; Woodhams et al. 2008); where possible we select approximately midway values. Additionally, sometimes we examine parameter values that differ from the default in order to more clearly demonstrate particular analytical findings (e.g., specific bifurcations) numerically.

**Table 1:** A summary of parameters/functions for the innate and learned behavioral avoidance models, and their selected default values

Name	Meaning	Default Value(s)	Constraint (s)
$\mu$	Birth/natural mortality	0.0006	$\mu >0$
$\beta$	Infection rate	Varied	$\beta >0$
$\alpha (P)$	Avoidance effectiveness function	Constant; $\alpha _0$ varied in (0, 1]	$0< \alpha (P) \le 1$, $\alpha'(P) \le 0$
*c*(*P*)	Survival cost of avoidance function	Constant; $c_0$ varied in {0, 0.25$\mu$, $\mu$}	$0 \le c(P) \le \phi$, $c'(P) \ge 0$
$\gamma$	Recovery rate from infection	0.04$^a$	$\gamma > 0$
$\phi$	Disease-induced mortality rate	Varied in {0, 0.0044, 0.04, 0.16}$^b$	$\phi \ge 0$
$\theta$	Pathogen release rate	1	$\theta >0$
$\lambda$	Pathogen death rate	0.24$^c$	$\lambda >0$
*p*	Probability of learning avoidance post infection*	1	$0 \le p \le 1$
*w*	Forgetfulness rate for learned avoidance behavior*	0	$w \ge 0$
*v*	Management rate for inducing learned avoidance*	0 or varied	$v \ge 0$

* The parameter is only in the learned avoidance model; other parameters are in both models

$^a$(Wilber et al. 2016)

$^b$(Rosenblum et al. 2010; Tobler and Schmidt 2010)

$^c$(Woodhams et al. 2008)

We let days be our unit of time (*t*). For the natural mortality rate, we select $\mu = 0.0006$ as the default value, which results in approximately 20% annual natural mortality. For the recovery rate, we select $\gamma = 0.04$, representing a median recovery period of about 17 days (equivalently, $\sim$10% chance of losing infection over a 3 day time step, reasonably within the range of moderate values given in Wilber et al. 2016). As the chances of dying from chytrid can vary from nearly nothing to nearly $100\%$ depending on species and environment (Rosenblum et al. 2010; Tobler and Schmidt 2010), we consider cases where the chance of dying from chytridiomycosis is absent, low (10%), medium (50%), and high (80%). Given our choice of $\gamma$, we thus select $\phi = 0$ (none), $\phi = 0.0044$ (low), $\phi = 0.04$ (medium), and $\phi = 0.16$ (high) for the disease-induced mortality rate.

We set $\lambda = 0.24$, representing a pathogen death rate of 0.01 per hour (Woodhams et al. 2008). The pathogen shedding rate $\theta$ controls the scaling of *P*, but since we are treating our state variables as unitless quantities, we fix $\theta =1$ for simplicity. To keep our simulations simple, we assume that learned avoidance behavior is not forgotten ($w = 0$) and that all infection events result in learning avoidance behavior ($p= 1$). For management effort to induce avoidance (*v*), we either assume it is absent ($v = 0$ indicates an ‘unmanaged’ system), or vary it over a wide interval to demonstrate a range of possible results. We also assume for simplicity in many of our simulations that behavioral avoidance is independent of pathogen concentration ($\alpha (P) = \alpha _0$ and $c(P) = c_0$), varying the effectiveness of behavior $\alpha _0$ in (0, 1] and the survival cost of behavior $c_0$ to represent an absent $(c_0 = 0)$, low ($c_0 = 0.25 \mu$), or high $(c_0 = \mu$) cost. However, we show some simulations with non-constant behavior in Sect. S1.8. Additionally, most of our analytical results hold in general for any choice of $\alpha (P), c(P)$ (that meets our previously noted constraints).

### General methods

To assist our analytical investigations of both models, we used the computer algebra software Maxima (version 5.47.0, Maxima 2023). All of these calculations are provided as Maxima scripts available on Figshare at https://doi.org/10.6084/m9.figshare.27914121 (Poulton and Ellner 2024). We also used the MatCont package (version 7.5, Dhooge et al. 2008) in MATLAB (version 24.1.0.2689473 (R2024a), The MathWorks Inc 2024) to carry out a numerical bifurcation analysis in Sect. 4.4. MatCont allows for the numerical identification and continuation (i.e., determination of how they move or change as parameter(s) are gradually varied) of equilibrium points, limit cycles, and most common types of bifurcations (Dhooge et al. 2008). Finally, simulations for both models were performed in R version 4.2.1 (R Core Team 2022), using the ode function with the Adams method from the deSolve package (Soetaert et al. 2010) to solve the system of ODEs. The data from the numerical bifurcation analysis in MatCont and the R scripts to recreate the simulations in Sect. 5 are also provided on Figshare (Poulton and Ellner 2024).

## Innate avoidance model analysis

### $R_0$ and disease-free equilibrium

Keeping in line with the biological interpretation of the innate avoidance model, we focus our analysis on the non-negative state space ($A, I, P \ge 0$). In Sect. S1.1, we formally define a positively invariant region ($B_{\text {innate}}$) for use in our analysis and show that so long as the initial conditions are non-negative, the state variables will remain non-negative for all time.

The disease-free equilibrium can be found by setting Eq. 1 equal to 0 with $I, P = 0$. The resulting disease-free equilibrium, DFE = ($\tilde{A}$, $\tilde{I}$, $\tilde{P}$), is given by

10 $$\begin{aligned} \tilde{A} = \frac{\mu }{\mu + c (0)}, \ \tilde{I} = 0, \ \tilde{P} = 0.\end{aligned}$$

We use the next generation matrix approach to calculate $R_0$ (van den Driessche and Watmough 2002). We begin by separating the dynamics for infectious compartments (*I*, *P*) into vectors representing the appearance of new infections ($\mathscr {F}$) and other transfers in/out of the compartment ($\mathscr {V}$),

11 $$\begin{aligned} \begin{bmatrix} I' \\ P' \end{bmatrix} = \mathscr {F} - \mathscr {V} = \begin{bmatrix} \alpha (P) \beta A P \\ 0 \end{bmatrix} - \begin{bmatrix} (\gamma +\phi +\mu ) I \\ \lambda P - \theta I \end{bmatrix}.\end{aligned}$$

Note that our decomposition of $\mathscr {F}, \mathscr {V}$ follows from our biological interpretation of the model, as we don’t consider additions to *P* to be new infections. Biological interpretations resulting in alternative valid $\mathscr {F}, \mathscr {V}$ decompositions may lead to different equations for $R_0$, although the threshold stability properties will be the same (Diekmann et al. 2010; van den Driessche and Watmough 2002). Let *F*, *V* be the Jacobians of $\mathscr {F}, \mathscr {V}$ evaluated at the DFE, respectively. Then $R_0$ is then equal to the spectral radius of $FV^{-1}$ (i.e., the maximum of the absolute values of the eigenvalues),

12 $$\begin{aligned} R_0 = \frac{ \alpha (0) \beta \theta \tilde{A } }{\lambda \left( \gamma + \phi + \mu \right) }.\end{aligned}$$

Note that this means we have $R_0 < 1$ when

13 $$\begin{aligned} \beta < \frac{\lambda \left( \gamma + \phi + \mu \right) }{ \alpha (0) \theta \tilde{A}} \end{aligned}$$

and vice versa. Equation 12 matches our previously stated definition for $R_0$, as an infected individual produces on average $\theta /(\gamma + \phi + \mu )$ units of pathogen, each of which causes (in an otherwise susceptible population) an average of $\alpha (0) \beta \tilde{A}/\lambda$ new infections. Equivalently to considering secondary cases of infected individuals, $R_0$ can also be interpreted in terms of pathogens (i.e., on average how many pathogens result from the infections caused by introducing a small unit of *P* to an otherwise susceptible population).

As our calculation of $R_0$ satisfies the conditions in Theorem A.1 of Diekmann et al. (2010), it follows that the disease-free equilibrium ($\tilde{A}, 0, 0$) is locally stable when $R_0 < 1$, and unstable for $R_0 > 1$. The same result can also be obtained directly by examining the eigenvalues of the Jacobian evaluated at the DFE, which we do in Maxima (IA_R0_DFEstability.wxmx; Poulton and Ellner 2024). We can further show that for $R_0 < 1$, the DFE is globally asymptotically stable in the positively invariant region $B_{\text {innate}}$ (Eq. S4). To do this, we follow the approach from Castillo-Chavez et al. (2002). This approach depends on showing two conditions: first, that in the reduced system with no disease, $\tilde{A}$ is globally asymptotically stable (H1), and second, that the dynamics for the infected compartments meet certain conditions stated below (H2). To check condition (H1), we set $I = P = 0$ in Eq. 1, which produces the reduced system $\frac{dA}{dt} = \mu - (\mu + c(0)) A$, or equivalently $\frac{dA}{dt} = - (\mu + c(0))(A - \tilde{A})$. Since this is just a linear differential equation and $\mu + c(0) > 0$, $\tilde{A}$ is globally asymptotically stable in the reduced system.

For condition (H2), we first write the dynamics for the infected compartments,

14 $$\begin{aligned} \begin{aligned} G =&\ \begin{bmatrix} \beta \alpha (P) AP - (\gamma + \mu + \phi ) I \\ \theta I - \lambda P \end{bmatrix} \, \end{aligned} \end{aligned}$$

and let the Jacobian of *G* evaluated at the DFE be denoted $DG_{I,P}(\tilde{A}, 0, 0)$. Then condition (H2) is that $\hat{G} = DG_{I,P}(\tilde{A}, 0, 0) [I, P]^{T} - G \ge 0$ for all $(A, I, P) \in B_{\text {innate}}$. Straightforward calculations show that

15 $$\begin{aligned} \begin{aligned} \hat{G} = \begin{bmatrix} \beta P( \alpha (0) \tilde{A} - \alpha (P) A) \\ 0 \end{bmatrix} \, \end{aligned} \end{aligned}$$

and thus $$\hat{G} \ge 0$$ is satisfied when $$ \alpha (0) \tilde{A} \ge \alpha (P) A $$ or $$ P= 0$$. Since $$A \le \tilde{A}$$ in $$B_{\text {innate}}$$ and $$\alpha'(P) \le 0$$, the condition (H2) is always satisfied in this region. Thus, the DFE is globally asymptotically stable in the region $$B_{\text {innate}}$$.

### Endemic equilibria

Here we solve for the endemic equilibria of the innate avoidance system (i.e., those equilibria with $I^*, P^* > 0$). Setting Eq. 3 equal to 0 and solving gives us $P^* = \frac{\theta }{\lambda }I^*$. Then, repeating the process for Eqs.1-2 and plugging in $P^* = \frac{\theta }{\lambda }I^*$ gives

16 $$\begin{aligned} A^* = \frac{\mu + \gamma I^*}{ \beta \alpha \left( \frac{\theta }{\lambda } I^*\right) \frac{\theta }{\lambda } I^* + \mu + c \left( \frac{\theta }{\lambda } I^*\right) } \ \ \text { and } \ \ A^* = \frac{ \gamma + \mu + \phi }{\frac{\theta }{\lambda }\beta \alpha \left( \frac{\theta }{\lambda } I^*\right) }.\end{aligned}$$

Solving further requires selecting forms for the behavioral functions $\alpha$ and *c*. Here we focus on the results for when $\alpha$ and *c* are constant functions. Letting $\alpha (P) = \alpha _0$ and $c(P) = c_0$, we find that there is a single endemic equilibrium $E^* = (A^*, I^*, P^*)$, given by

17 $$\begin{aligned} A^* = \frac{(\gamma +\phi +\mu ) \lambda }{\alpha _0 \beta \theta }, \ \ I^* = \frac{\mu \alpha _0 \beta \theta -(\mu +c_0)( \gamma +\phi + \mu ) \lambda }{(\phi +\mu ) \alpha _0 \beta \theta }, \ \ P^* = \frac{\theta }{\lambda }I^* .\end{aligned}$$

For this endemic equilibrium to exist (i.e., be positive), we must have $\beta > \frac{( \gamma +\phi + \mu ) \lambda }{ \alpha _0 \theta \tilde{A}}$, which is precisely the condition for $R_0 > 1$. Additionally, at $R_0 = 1$, the endemic equilibrium equals the DFE. By calculating the Jacobian and employing the Routh-Hurwitz criterion, we can show that the endemic equilibrium is locally stable for $R_0 > 1$, and undergoes a transcritical bifurcation with the DFE at $R_0 = 1$ (Sect. S1.2).

### Backward bifurcation

**Fig. 2:**
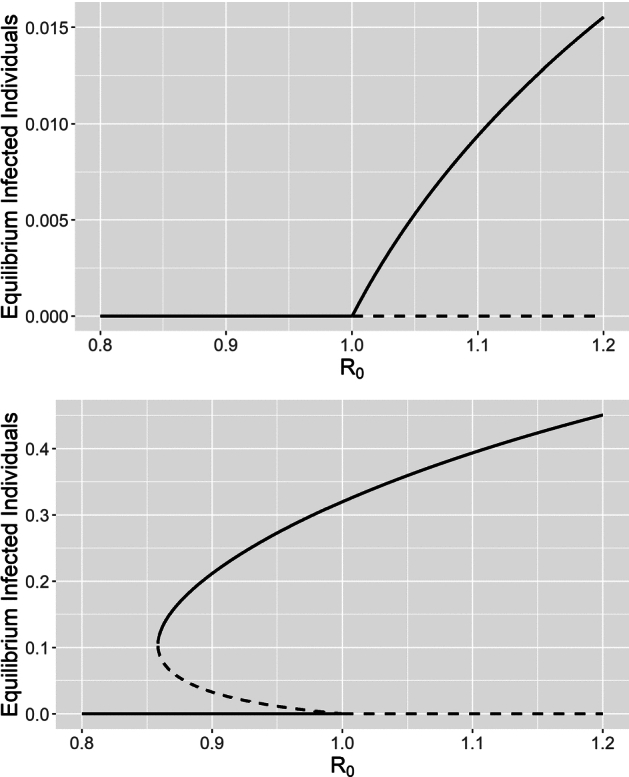
Examples from the learned avoidance system (see Sect.  4.3) of a forward transcritical bifurcation (top) and a backward transcritical bifurcation (bottom). In contrast, the transcritical bifurcation is always forwards in the innate avoidance system. Solid lines represent locally stable equilibria, while dashed lines represent unstable equilibria. Diagrams were generated by varying $\beta$, which has a linear relationship with $R_0$. Top: $\phi = 0.01$, bottom: $\phi = 0$. Other parameter values were $\alpha (P) = \alpha _0 = 0.2, c(P) = c_0 = 0, \mu = 0.0006, \gamma = 0.04, \theta = 1, \lambda = 0.24, p = 0.3, w = 0$ and $v = 0.005$

We previously showed that under constant behavioral functions $\alpha$ and *c*, the innate avoidance system has a single endemic equilibrium that exists (is positive) and is locally stable for $R_0 > 1$. This is also known as a ‘forward’ bifurcation, referring to the direction of the transcritical bifurcation at $R_0 = 1$ (Castillo-Chavez and Song 2004). The typical bifurcation diagram in this case as $R_0$ is varied looks similar to the top panel of Fig. 2. In this section, we show that a forward bifurcation occurs even when $\alpha$ and *c* are non-constant (so long as they follow the assumptions in Table 1). In contrast, another possibility is a ‘backward’ bifurcation, in which a positive endemic equilibrium emerges from the transcritical bifurcation to the left (Castillo-Chavez and Song 2004), as in the bottom panel of Fig. 2. This means that endemic equilibria exist, and the disease may persist, for $R_0 < 1$. Note that disease persistence also demonstrates hysteresis in this case, as the disease will cause an outbreak if $R_0$ increases above 1, but subsequently decreasing $R_0$ below 1 is not enough to ensure the disease dies out (e.g., in the bottom panel of Fig. 2, $R_0$ must decrease below $\approx 0.86$ to guarantee that the disease dies out). The examples in Fig. 2 are taken from the learned avoidance system (which we examine in detail in Sect. 4.3), and show that both such bifurcations are possible under learned avoidance.

Here we calculate the conditions for a backward bifurcation to occur, and show they cannot be satisfied under innate avoidance given our assumptions about the model; i.e., we show that the transcritical bifurcation is always forward. To do this, we use the approach in (Castillo-Chavez and Song 2004) (see also van den Driessche and Watmough 2002). The method, based on center manifold theory, allows us to not only show that a transcritical bifurcation of the DFE does occur at $R_0 = 1$, but also to calculate a general condition for the direction of this bifurcation. The calculations result in two bifurcation parameters, $\eta _1$ and $\eta _2$: a backward bifurcation occurs if and only if both of these are positive. The work for these calculations is lengthy and is provided as a Maxima script (IA_backwardsBifurcation.wxmx; Poulton and Ellner 2024). The resulting bifurcation parameters are given in Eqs. 18-19. For brevity, in these equations we let $\alpha = \alpha (0) > 0$, $c = c(0) \ge 0$, $\alpha' = \alpha'(0) \le 0$, and $c' = c'(0) \ge 0$.

18 $$\begin{aligned} \eta _1 =&\ - (\mu +c)^3 (\gamma +\phi +\mu ) \lambda (\mu \phi \alpha \lambda +c \phi \alpha \lambda +\mu ^2 \alpha \lambda +c \mu \alpha \lambda -\mu ^2 \alpha' \theta -c \mu \alpha' \theta +c' \mu \alpha \theta ) \, \end{aligned}$$

19 $$\begin{aligned} \eta _2 =&\ \mu (\mu +c) \alpha \theta.\end{aligned}$$

We can easily see that $\eta _2$ is always positive. However, $\eta _1$ is only positive if

20 $$\begin{aligned} \alpha' > \frac{\left( \left( \mu +c\right) \phi +{{\mu }^{2}}+c \mu \right) \alpha \lambda + c' \mu \alpha \theta }{\left( {{\mu }^{2}}+c \mu \right) \theta }.\end{aligned}$$

The right hand side of Eq. 20 is positive, so for a backward bifurcation to occur, we must at the very least have $\alpha'(0) > 0$; however, we assumed that under behavioral avoidance, $\alpha'(0) \le 0$. If this is the case, then $\eta _2$ must be negative, and the transcritical bifurcation at $R_0 = 1$ in the innate avoidance system is always forwards. Under a different model interpretation where $\alpha'(0) > 0$ is possible, though, a backward bifurcation could potentially occur; for instance, if $\alpha (P)$ included within it terms such that transmission was a nonlinear function of incidence. Past work has shown that nonlinear incidence rates can indeed lead to backward bifurcations, see for example Jin et al. (2007).

## Learned avoidance model analysis

### $R_0$ and disease-free equilibrium

We focus our analysis of the learned avoidance model on the non-negative state space ($S, A, I, P \ge 0$) to be consistent with biological interpretations. In Sect. S1.1, we again define a positively invariant region ($B_{\text {learned}}$) for use in our analysis and show that so long as the initial conditions are non-negative, the state variables will remain non-negative for all time.

Letting *I*, *P* = 0 and solving $\frac{dS}{dt}=0, \frac{dA}{dt} = 0$ for the learned avoidance system gives us the disease-free equilibrium, DFE = $(\tilde{S}, \tilde{A}, \tilde{I}, \tilde{P})$, where $\tilde{I},\tilde{P} = 0$ and

21 $$\begin{aligned} \tilde{S} = \frac{\mu (w + \mu + c(0))}{\mu (w + \mu + c(0)) + v(\mu + c(0))}, \ \ \tilde{A} = \frac{\mu v}{\mu (w + \mu + c(0)) + v(\mu + c(0))}.\end{aligned}$$

We again use the next generation matrix approach to calculate $R_0$ (van den Driessche and Watmough 2002). Separating the dynamics for infectious compartments (*I*, *P*) into vectors representing the appearance of new infections ($\mathscr {F}$) and other transfers in/out of the compartment ($\mathscr {V}$),

22 $$\begin{aligned} \begin{bmatrix} I' \\ P' \end{bmatrix} = \mathscr {F} - \mathscr {V}= \begin{bmatrix} \alpha (P) \beta A P +\beta P S \\ 0 \end{bmatrix} - \begin{bmatrix} (\gamma +\phi +\mu ) I \\ \lambda P -\theta I \end{bmatrix}.\end{aligned}$$

Again letting *F*, *V* be the Jacobians of $\mathscr {F}, \mathscr {V}$ evaluated at the DFE, $R_0$ is found by taking the spectral radius of $FV^{-1}$, from which we obtain

23 $$\begin{aligned} R_0 = \frac{\left( \alpha (0) \tilde{A} + \tilde{S}\right) \beta \theta }{\lambda \left( \gamma +\phi +\mu \right) }.\end{aligned}$$

In terms of $\beta$, we have that $R_0 < 1$ when

24 $$\begin{aligned} \beta < \frac{ \lambda \left( \gamma +\phi +\mu \right) }{\left( \alpha (0)\tilde{A} + \tilde{S}\right) \theta }.\end{aligned}$$

Note the similarity to the form of $R_0$ in the innate avoidance system: the only difference is that $\alpha (0) \tilde{A}$ in Eq. 12 has been replaced with $\alpha (0) \tilde{A} + \tilde{S}$ in Eq. 23. This quantity can be thought of as the ‘effective number’ of susceptible individuals in the disease-free equilibrium available to be infected (with $\tilde{A}$ scaled by $\alpha (0)$ due to the reduced chance of infection with avoidance behavior). As this quantity increases, $R_0$ also increases. Our calculation of $R_0$ again satisfies the conditions in Theorem A.1 of (Diekmann et al. 2010), and thus the disease-free equilibrium ($\tilde{A}, 0, 0$) is locally stable when $R_0 < 1$, and unstable for $R_0 > 1$. The same result can also be obtained by examining the eigenvalues of the Jacobian evaluated at the DFE, which we do in Maxima (LA_R0_DFEstability.wxmx; Poulton and Ellner 2024).

For the innate avoidance system, we showed that the DFE is globally asymptotically stable in the region $B_{\text {innate}}$ for $R_0 < 1$. This turns out to not always be the case under learned avoidance, as we show in the next two sections that a stable endemic equilibrium may exist for $R_0 < 1$. Despite this, there are certain subcases in which we can show that the DFE is globally asymptotically stable in the positively invariant region $B_{\text {learned}}$ (Eq. S5). In Sect. S1.3, we show that this is the case for the ‘unmanaged’ ($v = 0$) system, again using the approach from (Castillo-Chavez et al. 2002).

### Endemic equilibria

To solve for the endemic equilibria, we first set Eq. 8 equal to 0, which again gives us $P^* = \frac{\theta }{\lambda }I^*$. Then, repeating the process for Eqs. 5-7, we obtain

25 $$\begin{aligned} \begin{aligned} S^* =&\ \frac{\gamma + \mu + \phi -\beta \frac{\theta }{\lambda } \alpha \left( \frac{\theta }{\lambda }I^*\right) A^*}{\beta \frac{\theta }{\lambda }} \, \\ A^* =&\ \frac{p\gamma I^* + vS^*}{\mu + c\left( \frac{\theta }{\lambda }I^*\right) +\beta \frac{\theta }{\lambda } \alpha \left( \frac{\theta }{\lambda }I^*\right) I^* + w} \, \\ I^* =&\ \frac{\mu + wA^* - (v + \mu ) S^*}{\beta \frac{\theta }{\lambda }S^* - (1-p)\gamma }.\end{aligned} \end{aligned}$$

Just as for the innate avoidance system, solving further requires choosing forms for the behavioral functions $\alpha$ and *c*. Here we focus on the case of constant $\alpha$ and *c*. The resulting expressions for the endemic equilibria are long and are discussed further in Sect. S1.4. But the key result is that, depending on parameter values, either zero, one, or two endemic equilibria can exist (i.e., are real and positive). In the case of a forward bifurcation, one endemic equilibrium, which we denote as $E^* = (S^*, A^*, I^*, P^*)$, exists for $R_0 > 1$. In the case of a backward bifurcation, a saddle-node bifurcation at $R_0 = r^* <1$ results in two endemic equilibria (see Fig. 2 bottom, Sect. S1.4). The endemic equilibrium $E^*$ exists for $R_0 \ge r^*$, while the other endemic equilibrium (which we denote $E_2^*$, aka the ‘secondary’ endemic equilibrium), only exists for $r^* \le R_0 < 1$. Thus, much of our attention will be focused on the endemic equilibrium $E^*$.

In the innate avoidance model, the endemic equilibrium was always locally stable for $R_0 > 1$. However, this is not the case for the learned avoidance model: $E^*$ may be stable or unstable for $R_0 > 1$ (and similarly for $R_0 < 1$, when it exists). While the learned avoidance system is too complex to draw meaningful conclusions about local stability from eigenvalue conditions, in Sect. 4.4 we use the MatCont software to demonstrate that the local stability of $E^*$ may change through Hopf bifurcation(s).

While the endemic equilibrium $E^*$ may sometimes be unstable for $R_0 > 1$, we can show that the disease is uniformly persistent in the system when $R_0 > 1$. We define uniform disease persistence as follows (similar to LeJeune and Browne 2023): there exists an $\epsilon > 0$ such that for all initial conditions $(S_0, A_0, I_0, P_0)$ with $\min \{ I_0, P_0 \} >0$ in a region of interest, $\liminf _{t \rightarrow \infty } \ \min \{ I(t), P(t)\} > \epsilon$. We actually prove a more general conclusion, that all state variables are uniformly persistent for $R_0 > 1$. A proof of uniform persistence is given in Sect. S1.5, following the approach in (Freedman et al. 1994). The main step of the proof is to identify a closed positively invariant set *B* and show that the disease-free equilibrium is the maximal invariant set on the boundary of *B*. After checking a few additional conditions, the uniform persistence result for $R_0 > 1$ follows from simply noting that the disease-free equilibrium is unstable for $R_0 > 1$.

### Backward bifurcation

We again use the approach of Castillo-Chavez and Song (2004) to calculate the condition for the transcritical bifurcation at $R_0 = 1$ to be backwards. The calculation steps are provided as a Maxima script (LA_backwardsBifurcation.wxmx; Poulton and Ellner 2024) and hold for any choice of the behavioral functions $\alpha$ and *c*. The resulting bifurcation parameters $\eta _1$ and $\eta _2$ are given in Equations 26-28; a backward bifurcation occurs if and only if both of these are positive. For brevity, we let $\alpha = \alpha (0), c = c(0), \alpha' = \alpha'(0)$, and $c' = c'(0)$.

26 $$\begin{aligned} \eta _1 =&\ \kappa _1 (\kappa _2 \tilde{A} + \kappa _3 \tilde{S} + \kappa _4 \tilde{A}(\alpha \tilde{A} + \tilde{S})) \, \end{aligned}$$

27 $$\begin{aligned} \eta _2 =&\ \left( \mu w+\mu v+c v+{{\mu }^{2}}+c \mu \right) {{\left( \alpha \tilde{A} + \tilde{S}\right) }^{2}} \theta \, \end{aligned}$$

where

28 $$\begin{aligned} \begin{aligned} \kappa _1&= 2 (\mu w+\mu v+c v+\mu ^2+c \mu ) (\gamma +\phi +\mu ) \lambda \, \\ \kappa _2&= \alpha \lambda (\mu p \alpha \gamma -\mu \alpha \gamma -\mu p \gamma -c p \gamma +\mu \gamma +c \gamma -\mu v \alpha -\mu ^2 \alpha -\mu w -\phi (v \alpha +\mu \alpha +w ) ) \, \\ \kappa _3&= \lambda (\mu p \alpha \gamma -\mu p \gamma -c p \gamma -\mu v \alpha -\mu w -\mu ^2 -c \mu -\phi (v \alpha +w +\mu +c )) \, \\ \kappa _4&= \theta ( (\mu w +\mu v +c v +\mu ^2 +c \mu )\alpha' - (v \alpha +\mu \alpha +w )c' ).\end{aligned} \end{aligned}$$

**Fig. 3:**
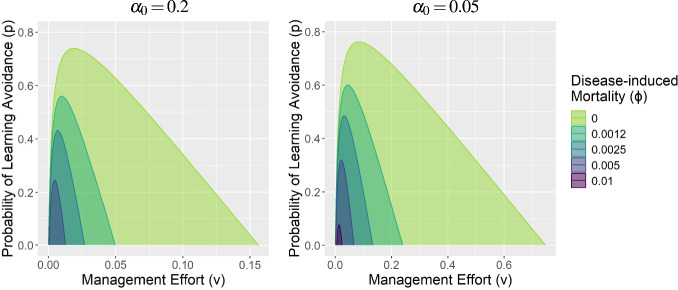
The regions of (*v*, *p*) parameter space for which a backward bifurcation occurs in the learned avoidance model as disease-induced mortality is varied. **Left:** $\alpha (P) = \alpha _0 = 0.2$, **right:** $\alpha (P) = \alpha _0 = 0.05$. The overlapping shaded areas for different values of $\phi$ represent where the transcritical bifurcation at $R_0 = 1$ is backwards; outside these regions, the bifurcation is forwards. Backward bifurcations occurred over larger areas of parameter space when behavioral avoidance was more effective (note the different x-axis scales). Other parameters have the values $c(P) = c_0 = 0, \mu = 0.0006, \gamma = 0.04, \theta = 1, \lambda = 0.24,$ and $w = 0$

It is easy to see that $\eta _2$ is always positive, but $\eta _1$ is more difficult to interpret. Here we make note of a few important properties of $\eta _1$. First, $\kappa _1$ is always positive, so we can ignore it and focus on the inner terms of $\eta _1$. Second, we wrote the formulas for $\kappa _2, \kappa _3$ in a specific way to emphasize the fact that increasing disease-induced mortality ($\phi$) decreases $\kappa _2$ and $\kappa _3$ (while having no effect on $\tilde{S}, \tilde{A}$, or $\kappa _4$). Thus, increasing $\phi$ decreases $\eta _1$, making a backward bifurcation ‘less likely’. If $\phi$ is sufficiently large, no backward bifurcation will occur. We demonstrate these observations in Fig. 3, which shows the regions of (*v*, *p*) space for which backward bifurcations occur for different values of $\phi$. With $\alpha _0 = 0.2$ (Fig. 3 left), backward bifurcations occur over a large area with $\phi = 0$, but the area shrinks rapidly as $\phi$ is increased, and by $\phi = 0.01$ backward bifurcations no longer occur. Note that given our chosen parameters, $\phi = 0.01$ corresponds to a $20\%$ mortality rate from each infection event; equivalently, it represents an approximately 16-fold increase in the mortality rate (compared to background natural mortality). When behavioral avoidance was more effective ($\alpha _0 = 0.05$ in the right panel of Fig. 3), backward bifurcations occurred for larger areas of (*v*, *p*) parameter space (note especially the change in the x-axis) and for slightly larger disease-induced mortality rates.

Third, again consider the unmanaged system ($v = 0$). Then $\tilde{S} = 1$, $\tilde{A} = 0$, and the condition for a backward bifurcation to occur becomes simply $\kappa _3 > 0$, or equivalently,

29 $$\begin{aligned} \alpha > \frac{ \mu p \gamma +c p \gamma +\mu w +{{\mu }^{2}} +c \mu + \phi (w +\mu +c ) }{\mu p \gamma }.\end{aligned}$$

The right hand side is greater than 1, which violates our assumptions about behavioral avoidance ($0< \alpha (P) \le 1$). Thus, backward bifurcations are not possible in the unmanaged system. This makes sense, as we previously showed that the DFE is globally asymptotically stable for $R_0 < 1$ in the unmanaged system. Finally, since we assumed that $\alpha' \le 0$ and $c' \ge 0$, we have that $\kappa _4\le 0$. This means that constant avoidance functions ($\alpha' = c' = 0$) represents a ‘best case’ scenario for a backward bifurcation to occur ($\kappa _4 = 0$). Under constant avoidance behavior, the condition for a backward bifurcation to occur is simply $\kappa _2 \tilde{A} + \kappa _3 \tilde{S} > 0$.

A backward bifurcation guarantees that an endemic equilibrium exists for some values of $R_0 < 1$, but it does not guarantee disease persistence for $R_0 < 1$. For one, there may or may not be a *locally stable* endemic equilibrium (or limit cycle), as we demonstrate in Sect. 4.4. Secondly, when there is a locally stable endemic equilibrium (or limit cycle), disease persistence depends on the initial conditions of the system — the dynamics are *bistable*. Trajectories that begin close enough to the DFE approach it (no disease persistence), while other trajectories approach the stable endemic equilibrium (or limit cycle). In rare cases, the bifurcation analysis presented below shows that the learned avoidance system can even demonstrate ‘tristability’, in which trajectories may approach the stable DFE, a stable endemic equilibrium, or a stable limit cycle.

Some intuition for the backward bifurcation can be gained by considering the ‘effective susceptible population size’, $\alpha (P(t)) A(t) + S(t)$. Consider starting from near the DFE ($\tilde{S}, \tilde{A}$) and introducing some amount of infection into the system. In typical cases, introducing infection causes a net flow from $S \rightarrow A$, as individuals survive infection and learn behavioral avoidance. This decreases the effective susceptible population size, and with fewer vulnerable individuals, the infection tends to die out. However, under certain parameter choices, introducing infection may actually cause a net flow from $A \rightarrow S$, leading to an increase in the effective susceptible population size. This can occur if there is management to induce learned avoidance ($v > 0$, meaning $\tilde{A} > 0$), but when individuals with learned avoidance become infected in the wild, they sometimes abandon the behavior upon recovery ($p < 1$ is sufficiently small). Then, if the initial infection is sufficiently large, the effective susceptible population size may increase to a level at which the infection can be sustained (see also (Reluga and Medlock 2007), who noted a similar finding). This observation also agrees with our findings regarding disease-induced mortality ($\phi$), as high enough disease-induced mortality leads to sufficient deaths in the population such that $\alpha (P(t)) A(t) + S(t)$ cannot increase to a level at which the infection can be sustained, no matter the flow between *A* and *S*.

### Limit cycles and bifurcations

We previously demonstrated that a transcritical bifurcation at $R_0 = 1$ results in the disease-free equilibrium switching stability, and that this bifurcation may be either forwards or backwards. In the case of a backward bifurcation, two endemic equilibria arise from a saddle-node bifurcation at some $R_0 < 1$. Both of these are codimension-1 bifurcations, meaning that they result as a single parameter is varied (Strogatz 2015). While these are the only two bifurcations in our system that change the number of equilibria that exist (i.e., that are real and positive), there are other codimension-1 bifurcations that change the stability of the equilibria and/or have other global effects, such as the creation/destruction of limit cycle(s). While it is possible to state general conditions for some of these bifurcations (like we did for the backward bifurcation), due to the complexity and number of parameters in our model, such conditions are impossible to interpret in a meaningful way. Thus, in this section we focus on using the MatCont software to numerically detect and continue these bifurcations. In these examples, we vary both the infection rate ($\beta$) and disease-induced mortality rate ($\phi$) while fixing the remaining parameters. Parameter values were selected to best demonstrate the resulting bifurcation curves, which sometimes only occur over very small areas.

**Fig. 4:**
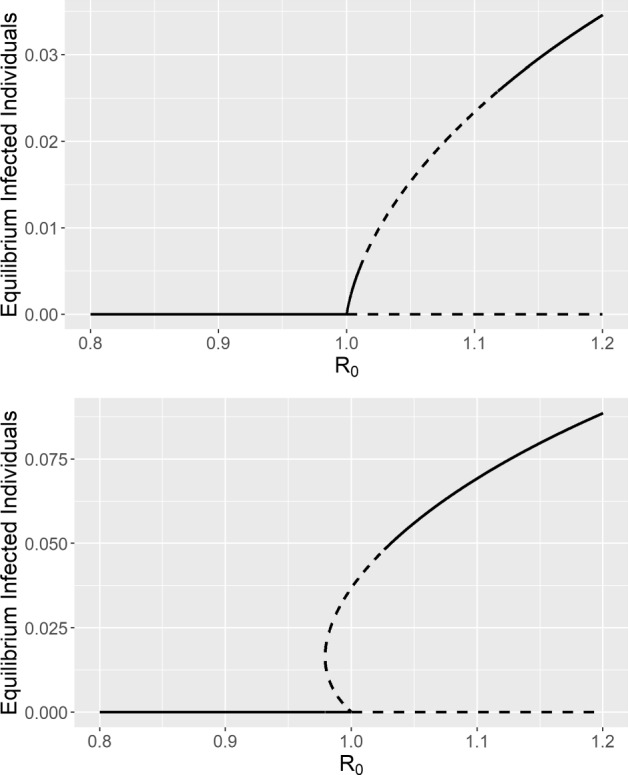
Two bifurcation diagrams demonstrating bifurcations of limit cycles in the learned avoidance model. Solid lines represent locally stable equilibria, while dashed lines represent unstable equilibria. Diagrams were generated by varying $\beta$ (which has a linear relationship with $R_0$). **Top** ($\phi = 0.005$): a supercritical Hopf bifurcation at $R_0 \approx 1.01$ results in the endemic equilibrium $E^*$ becoming unstable and a stable limit cycle appearing; another supercritical Hopf bifurcation at $R_0 \approx 1.12$ undoes this. **Bottom** ($\phi = 0.002$): a homoclinic bifurcation near $R_0 = 1$ results in the creation of a stable limit cycle, which is destroyed by a supercritical Hopf bifurcation at $R_0 \approx 1.03$. Parameter values used were $\alpha (P) = \alpha _0 = 0.2, c(P) = c_0 = 0, \mu = 0.0006, \gamma = 0.04, \theta = 1, \lambda = 0.24, p = 0.3, w = 0$ and $v = 0.005$.

**Fig. 5:**
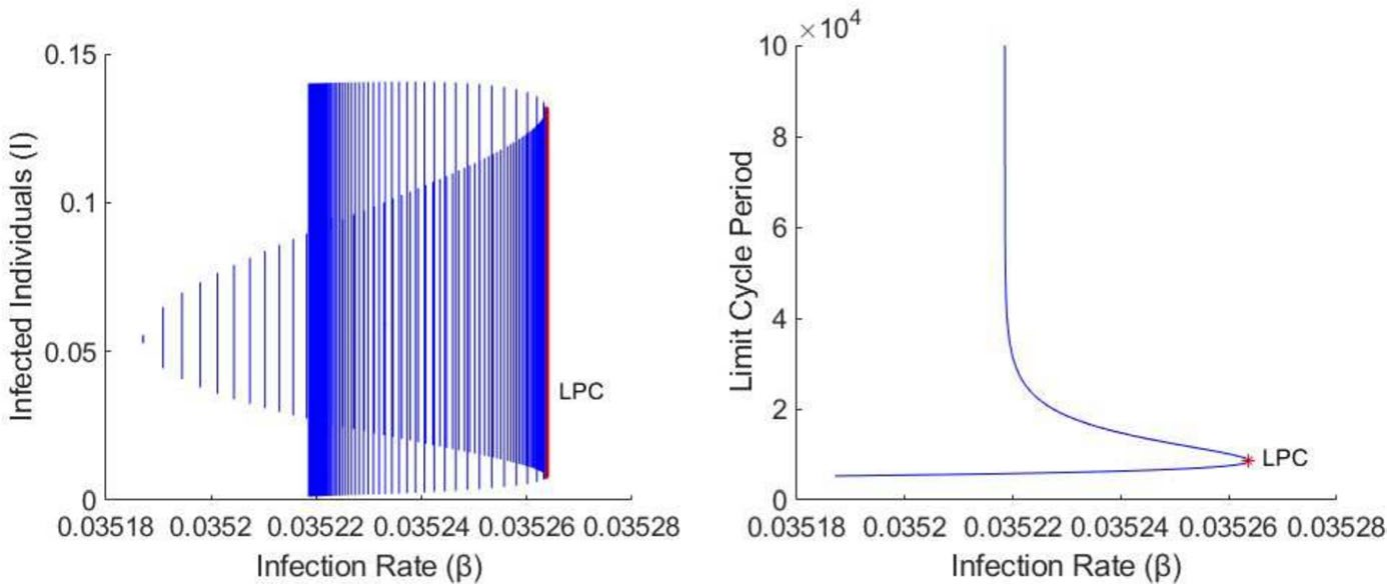
Demonstration of the three types of codimension-1 bifurcations of limit cycles in the learned avoidance system. The left plot shows the size (min/max number of infected individuals) of the resulting limit cycles(s), while the right plot shows the period of the limit cycle(s). As the infection rate $\beta$ increases (with $\phi = 0.0015$), a subcritical Hopf bifurcation results in an unstable limit cycle, a homoclinic bifurcation results in a stable limit cycle, and then the two limit cycles collide and disappear at the LPC (limit point bifurcation of cycles) point. Other parameters have the values $\alpha (P) = \alpha _0 = 0.2, c(P) = c_0 = 0, \mu = 0.0006, \gamma = 0.04, \theta = 1, \lambda = 0.24, p = 0.3, w = 0$ and $v = 0.005$.

A Hopf (i.e. Andronov-Hopf) bifurcation simultaneously results in the appearance of a limit cycle and the switch in stability of an equilibrium point (Strogatz 2015). In the learned avoidance system, we found that a Hopf bifurcation may occur around the endemic equilibrium $E^*$. The Hopf bifurcation may be either supercritical, resulting in a stable limit cycle and unstable fixed point, or subcritical, resulting in an unstable limit cycle and stable fixed point (Strogatz 2015). The top panel of Fig. 4 shows an example bifurcation diagram in which two supercritical Hopf bifurcations occur at $R_0 \approx 1.01$ and $R_0 \approx 1.12$; for $R_0$ in-between, the endemic equilibrium is unstable and a stable limit cycle exists. There is also a supercritical Hopf bifurcation in the bottom panel of Fig. 4 at $R_0 \approx 1.03$. In contrast, Fig. 5 shows an example in which there is a subcritical Hopf bifurcation that results in an unstable limit cycle.

We state the general condition for a Hopf bifurcation to occur at $E^*$ in Sect. S1.6. Due to the complexity our model, though, the resulting condition is difficult to derive meaning from. As for the other equilibria, we showed previously that the DFE only switches stability due to the transcritical bifurcation at $R_0 = 1$, and thus no Hopf bifurcations occur at the DFE. While the condition for a Hopf bifurcation to occur at the secondary endemic equilibrium $E_2^*$ is similarly difficult to interpret, numerical simulations support that $E_2^*$ is always unstable when it exists, and thus that there are no Hopf bifurcations around $E_2^*$ (Fig. S2).

A homoclinic bifurcation occurs when a limit cycle and saddle point collide, resulting in the destruction of the limit cycle (Strogatz 2015). This is a global bifurcation, and cannot be detected by examining the eigenvalues of the Jacobian at the equilibrium point (Strogatz 2015). As the limit cycle gets closer and closer to the saddle point, the period of the cycle approaches infinity; at the bifurcation point the limit cycle becomes a homoclinic orbit (Strogatz 2015). In numerical simulations of the learned avoidance model, we found that a homoclinic bifurcation could occur with the secondary endemic equilibrium $E_2^*$. Figure 5 shows an example in which the homoclinic bifurcation involves a stable limit cycle, while Fig. S3 shows an example in which it involves an unstable limit cycle. A homoclinic bifurcation also occurs in the bottom panel of Fig. 4 near $R_0 = 1$ (not observable via changes in fixed point stability), resulting in a stable limit cycle which is destroyed by a Hopf bifurcation as $R_0$ increases. The final codimension-1 bifurcation in our model is a limit point bifurcation of cycles (LPC), also known as a fold or saddle-node bifurcation of cycles. At this type of bifurcation, a stable and an unstable limit cycle collide and disappear (Kuznetsov 1998). This is another global bifurcation that cannot be detected through eigenvalue conditions of equilibria (Strogatz 2015). Figure 5 shows an example of an LPC bifurcation that occurs as the infection rate ($\beta$) varies. This figure also demonstrates that there are regions in the learned avoidance model in which a stable and unstable limit cycle coexist.

**Fig. 6:**
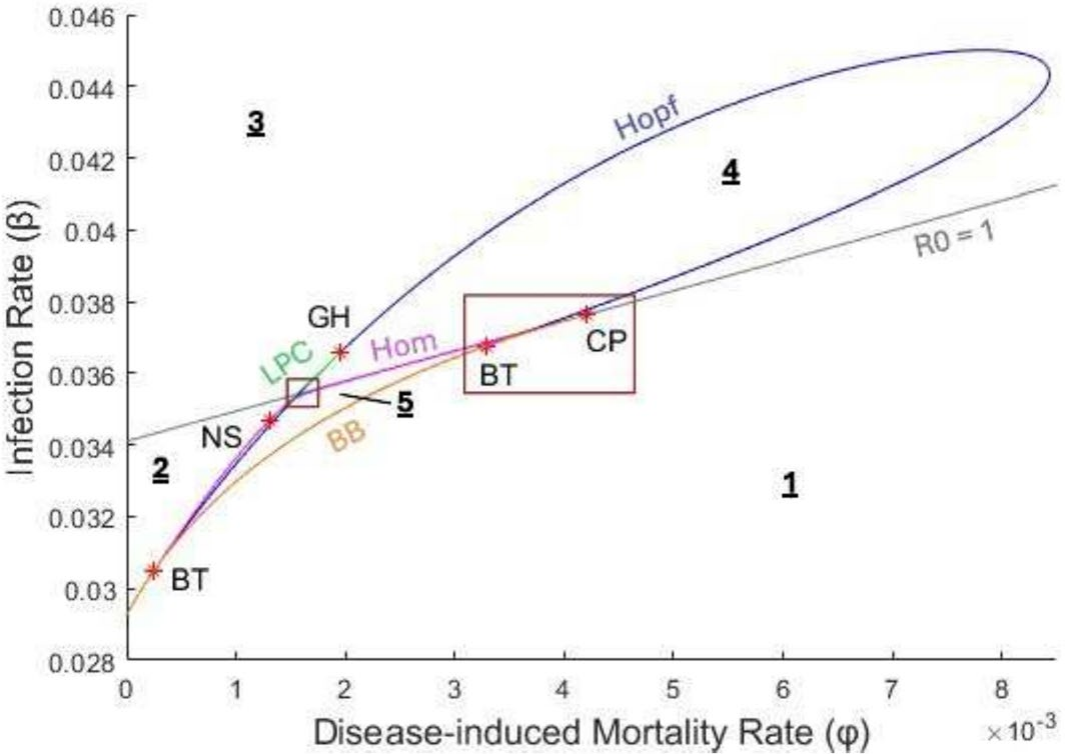
A bifurcation diagram of the learned avoidance system, varying the infection parameter $\beta$ and the disease-induced mortality parameter $\phi$. Only the five largest regions are indicated numerically here for clarity; zoomed in figures of the areas indicated with red boxes are given in Fig. 7. The grey line indicates the transcritical bifurcation at $R_0 = 1$, with the area above the line being $R_0 > 1$ and below $R_0 < 1$. Other bifurcation curves include a Hopf curve, the saddle-node curve arising from a backward bifurcation (BB), a homoclinic curve (Hom), and a limit point of cycles curve (LPC). Co-dimension two bifurcations are indicated with red stars and include two Bogdanov-Takens (BT) points, a cusp point (CP), a neutral saddle point (NS), and a generalized Hopf point (GH). The parameter values used were $\alpha (P) = \alpha _0 = 0.2, c(P) = c_0 = 0, \mu = 0.0006, \gamma = 0.04, \theta = 1, \lambda = 0.24, p = 0.3, w = 0$ and $v = 0.005$

**Fig. 7:**
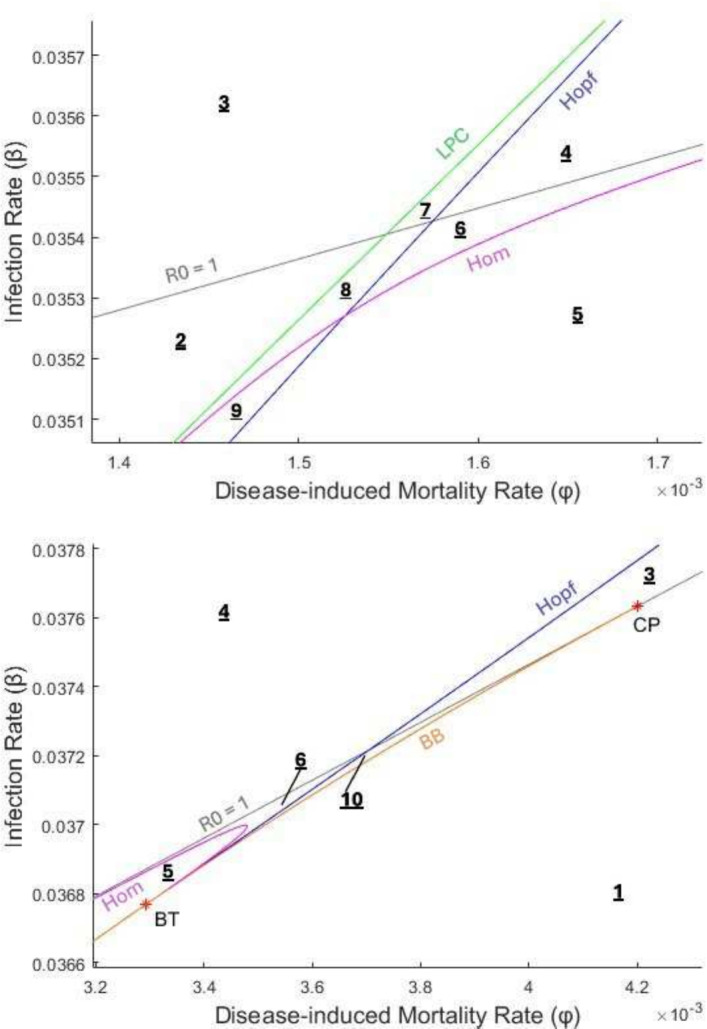
Zoomed in figures of the areas indicated by red boxes in Fig. 6 (**top**: left box, **bottom**: right box). The grey line indicates the transcritical bifurcation at $R_0 = 1$, with the area above the line being $R_0 > 1$ and below $R_0 < 1$. Other bifurcation curves include a Hopf curve, the saddle-node curve arising from a backward bifurcation (BB), a homoclinic curve (Hom), and a limit point of cycles curve (LPC). Co-dimension two bifurcations are indicated with red stars and include a Bogdanov-Takens point (BT) and a cusp point (CP). The parameter values used were the same as in Fig. 6

**Fig. 8:**
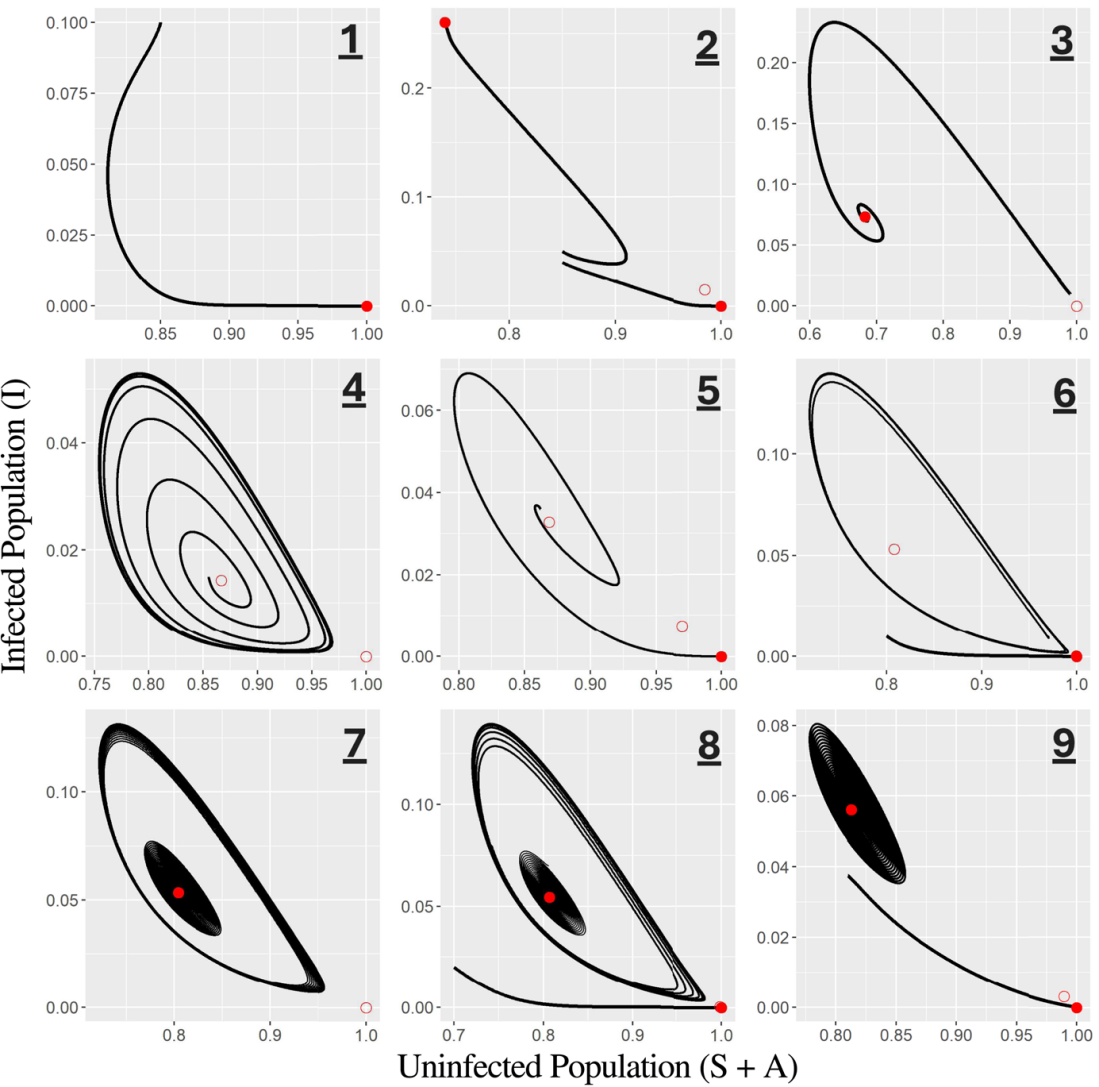
Sample trajectories corresponding to the labeled numeric regions in Figs. 6 and 7. Solid red dots represent (locally) stable equilibria, while open red dots represent unstable equilibria. Multiple trajectories are shown when bistability is present (multiple stable equilibria/cycles). Trajectories are described in detail in Table 2. 1) $\phi = 0.005, \beta = 0.035$. 2) $\phi = 0, \beta = 0.032$. 3) $\phi = 0.002, \beta = 0.04$. 4) $\phi = 0.005, \beta = 0.04$. 5) $\phi = 0.0018, \beta = 0.035$. 6) $\phi = 0.00157, \beta = 0.0354$. 7) $\phi = 0.0016, \beta = 0.035545$. 8) $\phi = 0.00153, \beta = 0.03533$. 9) $\phi = 0.0014, \beta = 0.0349$. Region 10 was qualitatively similar to region 2 and is not shown here. Line widths were varied to result in clearer depictions of each trajectory. Other parameter values were the same as in Fig.  6.

The five previously described codimension-1 bifurcations result in bifurcation curves as two parameters are varied. Figures 6 and 7 show a two-dimensional numerical bifurcation diagram for our model resulting from varying both the infection rate ($\beta$) and the disease-induced mortality rate ($\phi$). For instance, the backward bifurcation (BB) curve represents the values $(\phi, \beta )$ at which the saddle-node bifurcation of endemic equilibria occurs (and is positive). Similarly, the curve labeled $R_0 = 1$ shows where the transcritical bifurcation of the disease-free equilibrium occurs. The bifurcation curves divide the model into ten regions with distinct behavior. In Table 2, we describe for each region the existence/stability of equilibria, existence/stability of limit cycles, and typical behavior of trajectories. Sample trajectories for each region are shown in Fig. 8. For sample trajectories, we focus on the general behavior of the system towards attracting fixed points/limit cycles in the presence of disease (as we previously showed that if no disease is present, the DFE is always globally asymptotically stable).

As $\beta$ and $\phi$ are varied in Figs. 6 and 7, several types of codimension-2 bifurcations occur, which involve the creation/destruction of the codimension-1 bifurcation curves. A cusp point (CP) is found where the backward bifurcation curve collides with the line for the transcritical bifurcation at $R_0 = 1$, at approximately $(\phi, \beta ) = (0.004200, 0.037632$). The backward bifurcation curve ends at the cusp point, and thus the cusp point represents the transition between a backward bifurcation and forward bifurcation as $\phi$ increases. A Bodganov-Takens (BT) bifurcation occurs where a Hopf, homoclinic, and saddle-node curve meet, resulting in the end of the Hopf and homoclinic curves (Guckenheimer 1986). There are two BT points in Fig. 6, where the Hopf and homoclinic curves meet the backward bifurcation (i.e., saddle-node) curve at approximately $(\phi, \beta ) = (0.000246, 0.030474)$ and $(\phi, \beta ) = (0.003293, 0.036768)$. A neutral saddle (NS) point is found on the homoclinic curve near $(\phi, \beta ) =$ (0.001304, 0.034685). While a neutral saddle simply means that the Jacobian of an equilibrium point has two real eigenvalues that sum to zero, it sometimes indicates more interesting behavior; in this case, the neutral saddle point marks the start of the LPC curve (Guckenheimer 1986; Kuznetsov 1998). The LPC curve doesn’t exist for long, as nearby it collides with the Hopf curve and disappears at a generalized Hopf (GH) point at approximately $(\phi, \beta ) = (0.001948, 0.036564)$. A generalized Hopf (i.e., Bautin) bifurcation occurs when the Hopf bifurcation switches from supercritical to subcritical (Kuznetsov 1998). Thus, in Fig. 6 the Hopf bifurcation is subcritical for $\phi \lessapprox 0.001948$ and supercritical for $\phi \gtrapprox 0.001948$.

**Table 2:** A description of each numerically labeled region in Figs.6 and 7

Region	DFE	$E^*$	$E_2^*$	Limit Cycle(s)	Sample Trajectories
**1**	Stable				Trajectories approach the DFE
**2** (&** 10**)	Stable	Stable	Unstable		Bistability: trajectories approach the DFE or $E^*$
**3**	Unstable	Stable			Trajectories approach $E^*$
**4**	Unstable	Unstable		One (stable)	Trajectories approach the stable limit cycle
**5**	Stable	Unstable	Unstable		Trajectories approach the DFE
**6**	Stable	Unstable	Unstable	One (stable)	Bistability: trajectories approach the DFE or the stable limit cycle
**7**	Unstable	Stable		Two (stable & unstable)	Bistability: trajectories approach the stable limit cycle or slowly spiral towards $E^*$
**8**	Stable	Stable	Unstable	Two (stable & unstable)	Tristability: trajectories approach the DFE, the stable limit cycle, or slowly spiral towards $E^*$
**9**	Stable	Stable	Unstable	One (unstable)	Bistability: trajectories approach the DFE or slowly spiral towards $E^*$

Sample trajectories for each attracting equilibria/limit cycle are shown for each region in Fig. 8. Equilibrium points include the disease-free equilibrium (DFE), the endemic equilibrium $E^*$, and the ‘secondary’ endemic equilibrium $E_2^*$ (which only exists when a backward bifurcation occurs)

## Simulation results

**Fig. 9:**
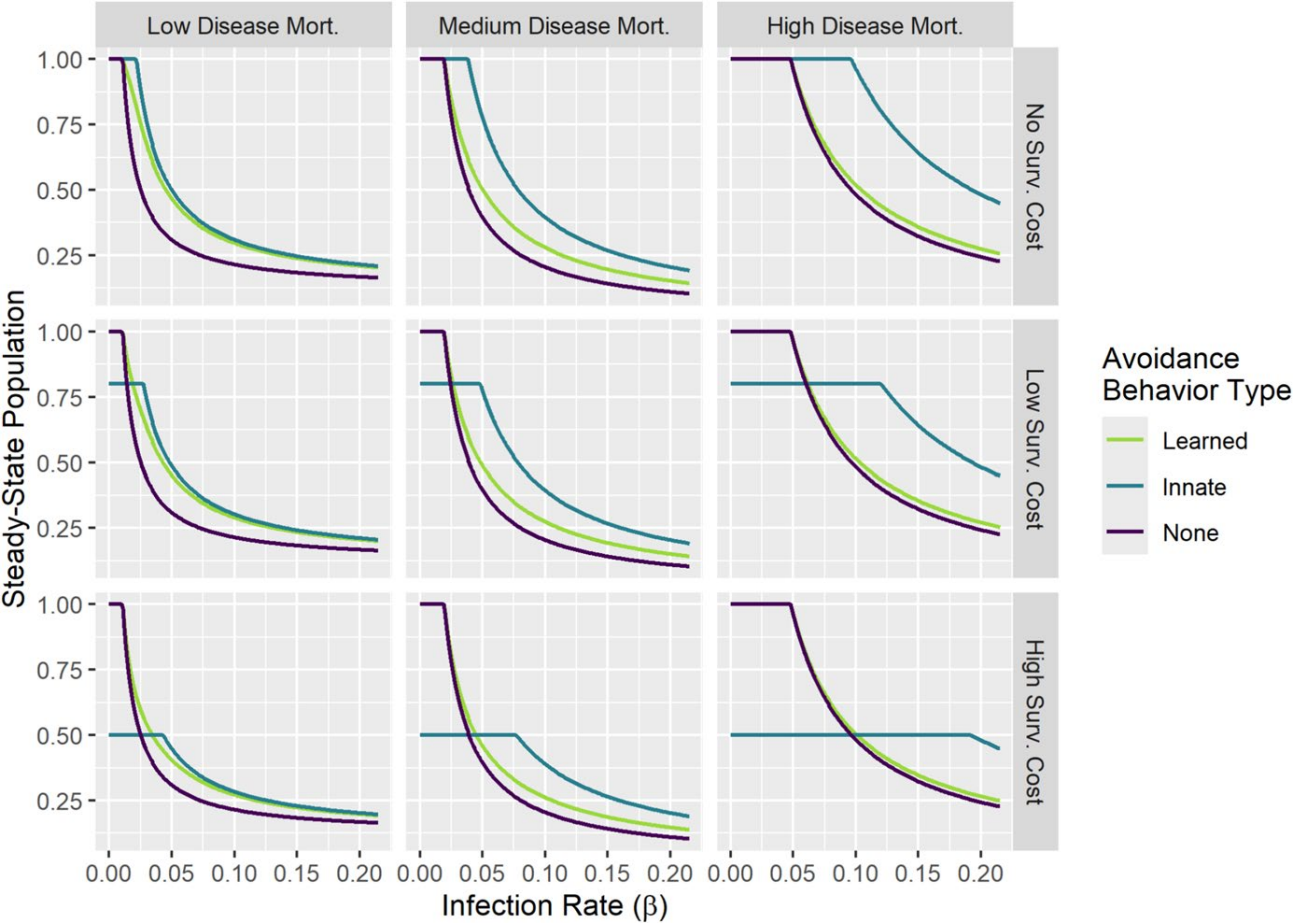
The steady-state population size resulting from learned, innate, or no behavioral avoidance as the infection rate ($\beta$) varies. Learned avoidance generally had similar outcomes to innate avoidance under low disease-induced mortality, and similar outcomes to no avoidance under high disease-induced mortality. Simulations assume constant avoidance behavior for the innate/learned avoidance models, with a moderate effectiveness of behavior ($\alpha (P) = \alpha _0 = 0.5$) and the survival cost $c(P) = c_0$ equal to 0 (no cost), $0.25\mu$ (low cost), or $\mu$ (high cost). The no avoidance model was obtained by setting $\alpha _0 = 1$ and $c_0 = 0$. Disease-induced mortality rates were $\phi = 0.0044$ (low), 0.04 (medium), or 0.16 (high). Other parameter values were $\mu = 0.0006, \gamma = 0.04, \theta = 1, \lambda = 0.24$, and for the learned avoidance model, $p = 1, w = 0, v = 0$.

In the presence of disease-induced mortality, avoidance behavior has the potential to increase host abundance, thus benefiting populations by decreasing the chance of extinction via demographic stochasticity. We begin by comparing the outcomes of innate versus learned avoidance behavior to examine what scenarios can lead one to outperform the other (in terms of increasing host population size), and how these outcomes compare to having no avoidance behavior. Assuming constant avoidance functions with $\alpha _0 = 0.5$ (and no management to induce learned avoidance), Fig. 9 shows the steady state population size as the infection rate varies in different mortality scenarios. We see that when disease-induced mortality is low, learned avoidance and innate avoidance generally have similar outcomes, with the steady-state population size being higher than under no avoidance. In the best case scenario, the steady-state population size under learned avoidance was more than $50\%$ greater than under no avoidance. The performance of learned and innate avoidance was even better when behavior was more effective (with $\alpha _0 = 0.25$ in Fig. S4, the steady-state population under learned avoidance was up to 127$\%$ greater than under no avoidance). With high disease-induced mortality, though, learned avoidance had similar outcomes to no avoidance. Even when behavioral avoidance was very effective, with the chance of dying from each infection event being 80%, few individuals had the chance to learn and benefit from avoidance. Unsurprisingly, the benefits of innate and learned avoidance behavior also lessened as the survival cost of avoidance increased.

While innate avoidance generally had the best outcomes, in some cases with a small infection rate it performed worse. This is because while all individuals possessing avoidance behavior prevented the disease from persisting, all individuals also experienced the survival cost of avoidance, which brought the steady-state population size down. In contrast, under learned avoidance if the disease cannot persist, the population eventually returns to all fully susceptible individuals (who don’t experience an extra survival cost). However, this difference may be rectified by examining non-constant avoidance behavior: for instance, a behavioral response that ‘turns off’ at very low pathogen concentrations (Figures S5 -S6).

**Fig. 10:**
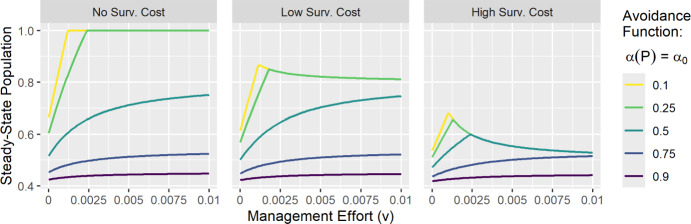
The steady-state population size resulting from varying levels of management effort to induce behavioral avoidance (*v*) in the learned avoidance system. Management had the most positive impact when avoidance behavior was effective (small $$\alpha _0$$) and the survival cost of avoidance was not too large. Simulations assume moderate disease-induced mortality ($$\phi = 0.04$$) and constant avoidance behavior, with the survival cost $$c(P) = c_0$$ equal to 0 (no cost), $$0.25\mu $$ (low cost), or $$\mu $$ (high cost). The infection rate $$\beta $$ was selected to result in $$R_0 = 2.5$$ when $$v = 0$$. Other parameter values were $$\mu = 0.0006, \gamma = 0.04, \theta = 1, \lambda = 0.24, p = 1,$$ and $$ w = 0$$.

We next consider the effectiveness of management effort to induce avoidance (*v*) in the learned avoidance system. Figure 10 shows the steady-state population size resulting from varying levels of management effort to induce learned avoidance. We found that management effort was most effective at increasing the steady-state population size when behavioral avoidance was strong, and the cost of behavioral avoidance was not too large. Steady-state population size was maximized at the point where management effort decreased $$ R_0$$ to 1 (note that backward bifurcations did not occur under the chosen simulated parameters). While increasing *v* further insured a greater decrease in $$R_0$$, in cases with a survival cost this leads to slightly decreasing steady-state population sizes. Further simulations showed that management effort was most effective under larger disease-induced mortality (Figure S7, top), which is intuitive since there is more to be gained by avoiding the disease when disease-induced mortality is high. As we assumed that management to induce behavior carries no mortality risk, it thus offers a safe path to learning avoidance behavior in the face of high disease-induced mortality. Management effort to induce avoidance was also more effective when the $$R_0$$ of the unmanaged system was closer to 1 (Figure S7, bottom).

## Discussion

We examined models representing two alternative mechanisms for behavioral avoidance: innate avoidance, which is present from birth, and learned avoidance, which may be gained after an infection event. The innate avoidance model showed relatively simple behavior, in which there was a single endemic equilibrium and disease persistence was only possible for $R_0 > 1$. In contrast, the learned behavioral avoidance model showed a wealth of codimension-1 and -2 bifurcations, resulting in potential disease persistence for $R_0 < 1$ and other complex model behavior, such as limit cycles. We provided analytical results for these bifurcations when possible, and otherwise performed numerical bifurcation analyses to demonstrate our observations. Our work showed that learned avoidance behavior may lead to interesting dynamics in waterborne (or more generally, environmentally-spread) disease systems, and that it may have unexpected consequences when interacting with management effort to control the disease. It is noteworthy that such varied and complicated dynamics are possible even for the simple models of avoidance behavior that we focused on in this paper.

Many management strategies to control the spread of diseases work by influencing factors to decrease the reproduction number (through vaccinations, quarantines, etc.), ideally bringing it below 1 (Ridenhour et al. 2014). Thus, backward bifurcations have important implications for disease management, as their existence in a system implies that bringing $R_0$ below one is not enough to ensure the disease dies out. Interestingly, backward bifurcations were only possible in the learned avoidance system, and only when management effort to induce avoidance behavior was non-zero. While this is an unexpected consequence, our analysis also demonstrated that backward bifurcations became ‘less likely’ under higher disease-induced mortality. As chytridiomycosis carries a high mortality risk for many species of amphibians (Rosenblum et al. 2010; Tobler and Schmidt 2010), backward bifurcations may be less of a concern for their management. However, we found that backward bifurcations were still possible at disease-induced mortality rates high enough to be relevant for many diseases of fish and wildlife.

Reintroductions of amphibians have failed due to Bd presence (Stockwell et al. 2011), which can persist in the environment and on more tolerant host species (Brannelly et al. 2015; Johnson and Speare 2005; Narayan et al. 2014). (McMahon et al. 2021) suggest that inducing avoidance behavior (e.g., in captive bred individuals before release) could help improve the establishment and persistence of amphibian populations. Our simulations of management effort to induce avoidance behavior showed that it could increase the size of amphibian populations and even reduce $R_0$ below 1, especially when the behavioral response was strong and with little additional survival cost. However, our work considered a relatively simple implementation of such management (constant effort over time) in wild individuals. Future work could consider time-varying management strategies, or even add an optimal control aspect to the model to consider when it is best to apply management effort to induce avoidance behavior. For instance, one might consider the optimization of a captive-breeding program in which behavior may be induced in individuals before release at an additional cost. Of course, in practice, management to induce behavior would only be useful in species which have the capacity for learning behavioral avoidance: while (McMahon et al. 2014, 2021) found two frog species that demonstrated significant learned avoidance, two other tested species did not demonstrate such a capacity. Behavioral studies, such as those performed in (McMahon et al. 2014, 2021), would be needed before implementing such a management strategy for other species.

We made some simplifications to our model to make obtaining analytical results more feasible, but there are many interesting questions that could be answered with more complex models of learned and innate behavioral avoidance. For one, our model assumes a constant environment through time, but in reality, seasonality is not only an important aspect of amphibian population dynamics (e.g. reproduction, migration) (Borzée et al. 2019; Kinney et al. 2011) but also Bd dynamics. The growth of Bd is maximized around 17-25 $^{\circ }$C (62-77 $^{\circ }$F), with slower or no growth outside this range (Piotrowski et al. 2004), meaning that prevalence and impacts of Bd may vary greatly over the course of a year (Kinney et al. 2011; Lenker et al. 2014; Longo et al. 2010). We also kept our learned avoidance model to four state variables, focusing on how avoidance might confer a lower chance of infection. However, should infection occur, avoidance could also potentially result in lower infection severity or faster recovery, in which case a model with multiple infectious compartments would be worth examining. Furthermore, we did not consider a load-dependent model, although in reality the impacts of Bd (e.g. mortality) are highly load-dependent (Wilber et al. 2016). A model with sequential infectious stages, or better yet an integral projection model (e.g., Wilber et al. 2016), would be best suited for analyzing the interactions between behavior and load. Other potential generalizations of our model include examining non-constant recruitment functions or additional modes of infection (e.g., direct host-to-host transmission along with environmental transmission).

Because we focused on relatively simple models for avoidance behavior, there is scope for further research examining more complicated adaptive behavioral responses. For instance, realistically, animals may abandon avoidance behaviors quicker if the cost is higher, when disease incidence is low, or when other mortality risks are more pressing (e.g., if avoidance behavior makes it harder to capture prey and they and they are close to starvation). Finally, an interesting question is that of the evolution of avoidance behavior. (McMahon et al. 2021) theorize that the lack of significant innate/learned avoidance in some tested species could be related to species having only a limited evolutionary history with Bd, or already having other resistance/tolerance mechanisms that result in a lower selective pressure for avoidance. Future modeling work could study what factors favor the evolution of avoidance behavior, and how it interacts with other resistance/tolerance mechanisms.

Disease outbreaks can cause increased pressures on animal populations already impacted by climate change, invasive species, habitat loss, and other anthropogenic pressures (Smith et al. 2009; Wake and Vredenburg 2008). Such factors may also increase the emergence and spread of wildlife diseases (Lafferty and Gerber 2002; Smith et al. 2009), potentially leading to worldwide impacts, as has been seen with chytridiomycosis (Scheele et al. 2019). Behavioral avoidance offers an additional route for animals to resist potentially deadly diseases, and has been demonstrated in a variety of taxa. Our work illustrates important dynamics related to innate and learned avoidance in waterborne diseases, and shows how and when management to induce avoidance behavior may help bolster host populations. Inducing behavior as a management tactic has indeed already proven successful in other contexts: for example, conditioned taste aversion has been used to dissuade threatened predators from consuming toxic prey (Ward-Fear et al. 2024) and predator avoidance behavior can be induced in captured/captive-bred prey to help improve survival after release (Edwards et al. 2021). Behavioral avoidance thus offers an additional option alongside a suite of existing disease management strategies in wildlife (Langwig et al. 2015) that could help improve future conservation and reintroduction efforts.

## Supplementary Information

Below is the link to the electronic supplementary material.

Supplementary file 1 (pdf 550 KB)

## Data Availability

Novel code, Maxima scripts, and output (Poulton and Ellner 2024) are available on Figshare (https://doi.org/10.6084/m9.figshare.27914121).
