# Supplementary material for: Learned behavioral avoidance can alter outbreak dynamics in a model for waterborne infectious diseases
Source: J Math Biol. 2025 Aug 18;91(3):28. doi: 10.1007/s00285-025-02252-7 (PMC12360992; doi:10.1007/s00285-025-02252-7)
Supplement: Supplementary file 1 — (pdf 550 KB) [file 285_2025_2252_MOESM1_ESM.pdf]

S1 Supplementary Materials

S1.1 Building Positively Invariant Regions

Innate Avoidance

We begin by noting that if the initial values for the state variables (A,I,P) are non-negative, then the state variables remain non-negative for all $t>0$:

$$\frac{dA}{dt}|_{A=0} = \mu+\gamma I>0, \quad \frac{dI}{dt}|_{I=0} = \beta\alpha(P)AP\geq 0, \quad \frac{dP}{dt}|_{P=0} = \theta I\geq 0\,. \tag{S1}$$

Furthermore, considering the total population size $(N=A+I)$, note that

$$\frac{dN}{dt}=\mu-\mu N-c(P)A-\phi I\leq\mu-\mu N-c(0)A-\phi I\leq\mu-(\mu+c(0))N\,, \tag{S2}$$

and thus,

$$\left.\frac{dN}{dt}\right|_{\mu/(\mu+c(0))}\leq 0\,. \tag{S3}$$

This means that if $N(0)\leq\frac{\mu}{\mu+c(0)}$, $N(t)\leq\frac{\mu}{\mu+c(0)}$ for all $t>0$. Combining this with our previous observations, we form the positively invariant region B_{innate}, which will be the focus of our analysis:

$$B_{\text{innate}}=\left\{(A,I,P)\mid A,I,P\geq 0 \text{ and } N=A+I\leq\frac{\mu}{\mu+c(0)}\right\}\,. \tag{S4}$$

Learned Avoidance

We again start by demonstrating that there is a positively invariant region for the model, which will be the focus of our analysis,

$$B_{\text{learned}} = \{(S, A, I, P) \mid S, A, I, P \geq 0 \text{ and } N = S + A + I \leq 1\} \,. \tag{S5}$$

The procedure to show B_{learned} is positively invariant follows that of the innate avoidance case. We first check that if the initial values for the state variables are non-negative, then the state variables remain non-negative for $t > 0$. It is clear from Equations 5-8 that $\frac{dS}{dt}|_{S=0}, \ \ldots \ , \frac{dP}{dt}|_{P=0}$ are all non-negative. Considering the total population size ($N = S + A + I$), we have that

$$\frac{dN}{dt}|_{N=1} = -c(P)A - \phi I \leq 0 \,. \tag{S6}$$

This means that if $N(0) \leq 1, N(t) \leq 1$ for all $t > 0$. Thus, the region B_{learned} is indeed positively invariant.

S1.2 Endemic Equilibrium of the Innate Avoidance System (Constant Avoidance)

The following stability analysis confirms that the endemic equilibrium (Equation 17) of the innate avoidance system with constant avoidance undergoes a transcritical bifurcation with the DFE at $R_0 = 1$, and is stable for $R_0 > 1$ (i.e., when it is positive).

The Jacobian evaluated at the endemic equilibrium is

$$J|_{E^*} = \begin{bmatrix} -\alpha_0\beta P^* - \mu - c_0 & \gamma & -\frac{(\gamma+\phi+\mu)\lambda}{\theta} \\ \alpha_0\beta P^* & -\gamma-\phi-\mu & \frac{(\gamma+\phi+\mu)\lambda}{\theta} \\ 0 & \theta & -\lambda \end{bmatrix} . \tag{S7}$$

The characteristic polynomial of the Jacobian is $x^3 + b_2x^2 + b_1x + b_0$, with

$$\begin{aligned} b_2 &= \lambda + \gamma + \alpha\beta P^* + \phi + 2\mu + c , \\ b_1 &= (\phi+\mu)\alpha\beta P^* + (\alpha\beta P^* + \mu + c)\lambda + (\mu + c)(\mu + \phi + \gamma) , \\ b_0 &= (\phi+\mu)\alpha\beta\lambda P^* . \end{aligned} \tag{S8}$$

By the Routh-Hurwitz criterion, all eigenvalues have negative real part if and only if $b_0, b_1, b_2 > 0$ and $b_1b_2 - b_0 > 0$. Since our parameters are non-negative, we see that all these coefficients are positive when $P^* > 0$, or equivalently $R_0 > 1$. Furthermore, since b_0 is the product of the first term in b_1 with the first term in b_2, we have that b_1b_2 is equal to b_0 plus a sum of terms that are all positive whenever $P^* > 0$; therefore, $b_1b_2 - b_0 > 0$ is also satisfied for $R_0 > 1$. This shows that all eigenvalues have negative real part, and thus the endemic equilibrium is locally stable, for $R_0 > 1$.

S1.3 Global Stability of the DFE in the Learned Avoidance System (No Management)

Here we show that in the 'unmanaged' ($v = 0$) learned avoidance system, the DFE is globally asymptotically stable in B_{learned} for $R_0 < 1$. We again check conditions (H1) and (H2) from Castillo-Chavez et al. 2002. First, we check that the DFE is globally asymptotically stable in the reduced system where $I = P = 0$:

$$\begin{bmatrix} S' \\ A' \end{bmatrix} = \begin{bmatrix} -v-\mu & w \\ v & -w-\mu-c(0) \end{bmatrix} \begin{bmatrix} S \\ A \end{bmatrix} + \begin{bmatrix} \mu \\ 0 \end{bmatrix}. \tag{S9}$$

The reduced system in Equation S9 is a nonhomogeneous linear system (which could be transformed into a homogeneous linear system by using the state variables $S-\tilde{S}$ and $A-\tilde{A}$). It is easy to see that the matrix in Equation S9 has negative trace and positive determinant, and thus $(\tilde{S},\tilde{A})$ is globally asymptotically stable for the reduced system.

For condition (H2), we again write the dynamics for the infected compartments,

$$G = \begin{bmatrix} \beta SP + \beta\alpha(P)AP - (\gamma+\mu+\phi)I \\ \theta I - \lambda P \end{bmatrix}. \tag{S10}$$

Let the Jacobian of G evaluated at the DFE be denoted $DG_{I,P}(\tilde{S},\tilde{A},0,0)$. Condition (H2) is that $\hat{G} = DG_{I,P}(\tilde{S},\tilde{A},0,0)[I,P]^T - G \geq 0$ for all $(S,A,I,P) \in B_{\text{learned}}$. Straightforward calculations show that

$$\hat{G} = \begin{bmatrix} \beta P(\tilde{S}+\alpha(0)\tilde{A}-S-\alpha(P)A) \\ 0 \end{bmatrix}. \tag{S11}$$

To have $\hat{G} \geq 0$, we must have $P=0$ or $S+\alpha(P)A \leq \tilde{S}+\alpha(0)\tilde{A}$ for all $(S,\ A,\ I,\ P) \in B_{\text{learned}}$. For the unmanaged system ($v=0$), the disease-free equilibrium is $\tilde{S}=1, \tilde{A}=0$, and the latter condition becomes simply $S+\alpha(P)A \leq 1$. This is always satisfied in B_{learned}, since $N = S+A+I \leq 1$. Thus, for the learned avoidance system with no management ($v=0$), the DFE is globally asymptotically stable in B_{learned} for $R_0 < 1$.

S1.4 Endemic Equilibria of the Learned Avoidance System (Constant Avoidance)

For the learned behavioral avoidance system, we use Maxima to solve for the endemic equilibria $E^* = (S^*, A^*, I^*, P^*)$ and $E_2^* = (S_2^*, A_2^*, I_2^*, P_2^*)$ under the assumption that $\alpha(P)$, $c(P)$ are constant functions (α_0, c_0). The full expressions are quite lengthy and are given in a Maxima script (`LA_endemicEquilibria.wxmx`; Poulton and Ellner 2024), but here we make note of a few important properties. In particular, we can show that I^*, I_2^* are solutions to the quadratic equation $a_1x^2 + a_2x + a_3 = 0$,

$$I^* = \frac{-a_2 + \sqrt{a_2^2 - 4a_1a_3}}{2a_1}, \quad I_2^* = \frac{-a_2 - \sqrt{a_2^2 - 4a_1a_3}}{2a_1}, \tag{S12}$$

with

$$\begin{aligned}
a_1 &= (\phi+\mu)\alpha_0\beta\theta\,, \\
a_2 &= -((((\mu p-\mu)\alpha_0+(-\mu-c_0)p)\gamma+((-v-\mu)\phi-\mu v-\mu^2)\alpha_0 \\
&\quad +(-w-\mu-c_0)\phi-\mu w-\mu^2-c_0\mu)\lambda)-\mu\alpha_0\beta\theta\,, \\
a_3 &= (((\mu w+(\mu+c_0)v+\mu^2+c_0\mu)\gamma+(\mu w+(\mu+c_0)v+\mu^2+c_0\mu)\phi+\mu^2 w \\
&\quad +(\mu^2+c_0\mu)v+\mu^3+c_0\mu^2)\lambda^2+(-(\mu v\alpha_0)-\mu w-\mu^2-c_0\mu)\beta\theta\lambda)/(\beta\theta)\,.
\end{aligned} \tag{S13}$$

It can similarly be shown that the pairs S^*, S_2^* and A^*, A_2^* are also solutions to quadratic equations. Equation S12 has important implications for our study of the backward bifurcation, as the value $R_0 = r^*$ at which the saddle-node bifurcation of E^*, E_2^* occurs can be found by solving $a_2^2 - 4a_1a_3 = 0$ for β. Note that the saddle-node bifurcation may be positive ($I^*, I_2^* > 0$ when they appear), e.g. the bottom panel of Figure 2, or negative ($I^*, I_2^* < 0$ when they appear), e.g. Figure S1. This is determined by the sign of a_2 at $R_0 = r^*$ (since a_1 is always positive).

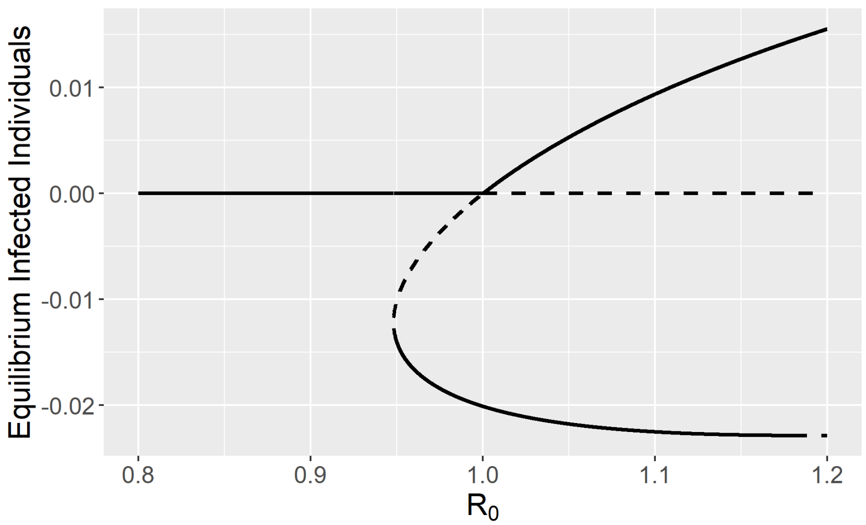

Figure S1: An expanded version of the bifurcation diagram shown in the top panel of Figure 2, showing the equilibria even when they are negative. The saddle-node bifurcation is negative (i.e., I^* and $I_2^* < 0$ when they appear). Solid lines represent locally stable equilibria, while dashed lines represent unstable equilibria. Parameter values were $\phi = 0.01, \alpha(P) = \alpha_0 = 0.2, c(P) = c_0 = 0, \mu = 0.0006, \gamma = 0.04, \theta = 1, \lambda = 0.24, p = 0.3, w = 0$ and $v = 0.005$.

The quadratic forms of I^*, I_2^* reveal several important properties about the endemic equilibria. First, we show in Maxima that $a_3 = 0$ if and only if $R_0 = 1$. This means that either I^* or I_2^* must equal 0 at $R_0 = 1$, depending on the sign of a_2 at $R_0 = 1$:

$$I^* = \frac{-a_2 + \sqrt{a_2^2}}{2a_1}\ , \quad I_2^* = \frac{-a_2 - \sqrt{a_2^2}}{2a_1} \quad \text{when } R_0 = 1\ . \tag{S14}$$

Thus, at $R_0 = 1$, one of the endemic equilibria crosses the DFE, resulting in a transcritical bifurcation. Since $R_0 = 1$ is the only time at which $a_3 = 0$, there can be no further changes in the signs of I^* or I_2^*, and therefore no further transcritical bifurcations. Furthermore, we show in Maxima that $a_3 < 0$ for $R_0 > 1$, from which we can conclude that I^* is positive and I_2^* is negative for $R_0 > 1$. For example, concerning

I_2^*, $R_0 > 1$ implies that

$$\begin{aligned} a_2^2 - 4a_1a_3 &> a_2^2 \,, \\ \sqrt{a_2^2 - 4a_1a_3} &> \sqrt{a_2^2} \geq -a_2 \,, \\ -a_2 - \sqrt{a_2^2 - 4a_1a_3} &< 0 \,, \\ I_2^* = \frac{-a_2 - \sqrt{a_2^2 - 4a_1a_3}}{2a_1} &< 0 \,. \end{aligned} \tag{S15}$$

Summarizing our conclusions, if the saddle-node bifurcation is positive, then the transcritical bifurcation at $R_0 = 1$ must involve the secondary endemic equilibrium E_2^*, which becomes negative for $R_0 > 1$ (i.e., a backward bifurcation). Similarly, if the saddle-node bifurcation is negative, then the transcritical bifurcation at $R_0 = 1$ must involve the endemic equilibrium E^*, which becomes positive for $R_0 > 1$ (i.e., a forward bifurcation).

S1.5 Uniform Persistence of the Learned Avoidance System

To prove uniform persistence, we follow the approach of Freedman et al. 1994, who give the following definition for uniform persistence: let a continuous flow (with continuous map $\pi(x,t)$) be defined on a metric space X with metric d, and let B be a closed, positively invariant subset of X with non-empty boundary (δB) and interior (B°). Then the flow is said to be uniformly persistent if there exists some $\varepsilon > 0$ such that $\forall x \in B^\circ$,

$$\liminf_{t\to\infty} d(\pi(x,t), \delta B) > \varepsilon \,. \tag{S16}$$

Our proof uses Theorem 4.3 of Freedman et al. 1994. The first step of the proof is to identify a closed, positively invariant set B for our system. Then, we show that the maximal invariant set (i.e., an invariant set that contains all other invariant sets) of the flow on δB is simply $\{\tilde{E}\}$, where $\tilde{E} = (\tilde{S}, \tilde{A}, 0, 0)$ is the

disease-free equilibrium. This can be accomplished by showing that trajectories that begin on δB either approach $\tilde{E}$ (in the case of $I_0 = P_0 = 0$) or leave and never reenter δB (in the case of $\min\{I_0, P_0\} > 0$). Being a set consisting of a single point, $\{\tilde{E}\}$ satisfies the conditions of hypothesis (H) in Freedman et al. 1994. Since we have already shown that the disease-free equilibrium is unstable for $R_0 > 1$, it is simple to show the remaining conditions in Theorem 4.3, from which we can conclude uniform persistence.

We previously noted that the region B_{learned} (Equation S5) is positively invariant for the learned avoidance system. For convenience in this proof, however, we define a slightly different positively invariant region, given by

$$\hat{B}_{\text{learned}} = \{(S,A,I,P) \mid S,A,I,P \geq 0,\ P \leq 2\theta/\lambda,\ \text{and}\, N = S+A+I \leq 2\}\ . \tag{S17}$$

We first check that this new set $\hat{B}_{\text{learned}}$ is indeed positively invariant. We previously showed that the state variables stay non-negative if they begin with non-negative initial conditions, so it is only necessary to check the two remaining boundary conditions. For total population size, $\frac{dN}{dt}|_{N=2} = -\mu - c(P)A - \phi I < 0$, and thus if $N_0 \leq 2$, $N(t) \leq 2$ for all t. Similarly for P, we find that $P_0 \leq 2\theta/\lambda$ implies $P(t) \leq 2\theta/\lambda$ because $\frac{dP}{dt}|_{P=2\theta/\lambda} = \theta I - 2\theta \leq 2\theta - 2\theta = 0$. Thus, $\hat{B}_{\text{learned}}$ is a positively invariant closed set.

Let δB be the boundary of $\hat{B}_{\text{learned}}$ and B° be the interior of $\hat{B}_{\text{learned}}$. Note that the disease-free equilibrium, $\tilde{E} = (\tilde{S}, \tilde{A}, 0, 0)$, falls on δB. Our next step is to show that the maximal invariant set on δB is $\{\tilde{E}\}$. We previously showed that with $I_0 = P_0 = 0$, $\tilde{E}$ is globally asymptotically stable. Furthermore, since $\hat{B}_{\text{learned}}$ is positively invariant, its interior B° is also positively invariant (e.g., Theorem 1.2.40 of Bhatia and Szegö 1967). This means that trajectories do not re-enter δB once they leave it. It remains to show that for $R_0 > 1$, any solution that begins on δB with $\min\{I_0, P_0\} > 0$ will leave δB. (This is why we defined $\hat{B}_{\text{learned}}$ as we did: had we used the boundary condition $S+A+I \leq 1$, then when $\phi = c(P) = 0$,

the endemic equilibrium would also fall on δB.)

Let the initial condition be $(S_0, A_0, I_0, P_0) \in \delta B$ with $\min\{I_0, P_0\} > 0$. It can be shown for each state variable that once it becomes positive, it remains positive for all time. (E.g., $\frac{dP}{dt} \geq -\lambda P$, which has solution $\propto e^{-\lambda t} > 0$). Again note that $\frac{dS}{dt}|_{S=0} > 0$, so if $S_0 = 0$ then $S(t)$ is positive for $t > 0$. Then consider the three following possible cases based on the initial values, which must satisfy $\min\{I_0, P_0\} > 0$.

- If $I_0, P_0 > 0$, then $I(t), P(t) > 0$ for all $t > 0$ since they must remain positive.
- If $P_0 = 0$, then $I_0 > 0$ and $\frac{dP}{dt}|_{P=0} = \theta I > 0$. Thus, $P(t) > 0$ for all $t > 0$.
- If $I_0 = 0$, then $P_0 > 0$ and $\frac{dI}{dt}|_{I=0} \geq \beta SP > 0$. Thus, $I(t) > 0$ for all $t > 0$.

Finally, given these results, $\frac{dA}{dt}|_{A=0} = p\gamma I + \nu S > 0$ shows that additionally if $A_0 = 0$ then $A(t) > 0$ for all $t > 0$. Next, we check the remaining two boundaries of $\hat{B}_{\text{learned}}$ to make sure that solutions leave δB. Since $\hat{B}_{\text{learned}}$ is positively invariant region satisfying $S + A + I \leq 2$ and $S(t) > 0$, we must have $I(t) < 2$ for $t > 0$. If $P_0 = 2\theta/\lambda$, then $\frac{dP}{dt}|_{P=2\theta/\lambda} = \theta(I - 2) < 0$ implies $P(t) < 2\theta/\lambda$ for $t > 0$. Similarly, if $N_0 = S_0 + A_0 + I_0 = 2$, then $\frac{dN}{dt}|_{N=2} = -\mu - c(P)A - \phi I < 0$ implies $N(t) < 2$ for all $t > 0$.

Therefore, if $(S_0, A_0, I_0, P_0) \in \delta B$ with $\min\{I_0, P_0\} > 0$, the solution will leave δB. It follows that $\{\tilde{E}\}$ is the maximal invariant set on δB. Being a single point, the set $\{\tilde{E}\}$ easily satisfies all conditions of hypothesis (H) of Freedman et al. 1994. Additionally, letting $N = \hat{B}_{\text{learned}}$ and $M = B^\circ \cap \mathrm{S}[\delta B, \varepsilon]$ (the set of points on the interior of B within ε distance of the boundary) in Definition 2.5 of Freedman et al. 1994, we see that the flow is point dissipative over $B^\circ \cap \mathrm{S}[\delta B, \varepsilon]$. Finally, Theorem 4.3 of Freedman et al. 1994 says that with the previously noted conditions met, the flow is uniformly persistent if and only if for all $x \in B^\circ \cap \mathrm{S}[\delta B, \varepsilon]$, $\omega(x) \not\subset \{\tilde{E}\}$, where ω indicates the omega limit set. This condition holds when the disease-free equilibrium $\tilde{E}$ is unstable, which we previously showed occurs for $R_0 > 1$. Therefore, the system is uniformly persistent when $R_0 > 1$.

S1.6 Hopf Bifurcations in the Learned Avoidance System

Using Maxima (`LA_hopf.wxmx`; Poulton and Ellner 2024), we show that the characteristic polynomial of the Jacobian evaluated at the endemic equilibrium E^* is $x^4 + b_1x^3 + b_2x^2 + b_3x + b_4$, where

$$
\begin{aligned}
b_1 =\;& \lambda+\gamma+P^*\alpha\beta+P^*\beta+\phi+w+v+3\mu+c\ ,\\
b_2 =\;& \gamma\lambda+P^*\alpha\beta\lambda+P^*\beta\lambda+\phi\lambda+w\lambda+v\lambda+3\mu\lambda+c\lambda-A^*P^*\alpha'\beta\theta-A^*\alpha\beta\theta-S^*\beta\theta-P^*p\alpha\beta\gamma\\
&+P^*\alpha\beta\gamma+P^*p\beta\gamma+w\gamma+v\gamma+2\mu\gamma+c\gamma+P^{*2}\alpha\beta^2+P^*\phi\alpha\beta+P^*v\alpha\beta+2P^*\mu\alpha\beta+P^*\phi\beta+P^*w\beta\\
&+2P^*\mu\beta+P^*c\beta+w\phi+v\phi+2\mu\phi+c\phi+2\mu w+2\mu v+cv+3\mu^2+2c\mu\ ,\\
b_3 =\;& -(P^*p\alpha\beta\gamma\lambda)+P^*\alpha\beta\gamma\lambda+P^*p\beta\gamma\lambda+w\gamma\lambda+v\gamma\lambda+2\mu\gamma\lambda+c\gamma\lambda+P^{*2}\alpha\beta^2\lambda+P^*\phi\alpha\beta\lambda+P^*v\alpha\beta\lambda\\
&+2P^*\mu\alpha\beta\lambda+P^*\phi\beta\lambda+P^*w\beta\lambda+2P^*\mu\beta\lambda+P^*c\beta\lambda+w\phi\lambda+v\phi\lambda+2\mu\phi\lambda+c\phi\lambda+2\mu w\lambda+2\mu v\lambda\\
&+cv\lambda+3\mu^2\lambda+2c\mu\lambda-A^*P^{*2}\alpha'\beta^2\theta-P^*S^*\alpha\beta^2\theta-A^*P^*\alpha\beta^2\theta-A^*P^*w\alpha'\beta\theta-A^*P^*v\alpha'\beta\theta\\
&-2A^*P^*\mu\alpha'\beta\theta-A^*P^*c\alpha'\beta\theta-A^*w\alpha\beta\theta-A^*v\alpha\beta\theta-2A^*\mu\alpha\beta\theta+A^*P^*c'\alpha\beta\theta-A^*c\alpha\beta\theta-S^*w\beta\theta\\
&-S^*v\beta\theta-2S^*\mu\beta\theta-S^*c\beta\theta-P^*\mu p\alpha\beta\gamma+P^*\mu\alpha\beta\gamma+P^*\mu p\beta\gamma+P^*cp\beta\gamma+\mu w\gamma+\mu v\gamma+cv\gamma+\mu^2\gamma\\
&+c\mu\gamma+P^{*2}\phi\alpha\beta^2+P^{*2}\mu\alpha\beta^2+P^*v\phi\alpha\beta+P^*\mu\phi\alpha\beta+P^*\mu v\alpha\beta+P^*\mu^2\alpha\beta+P^*w\phi\beta+P^*\mu\phi\beta\\
&+P^*c\phi\beta+P^*\mu w\beta+P^*\mu^2\beta+P^*c\mu\beta+\mu w\phi+\mu v\phi+cv\phi+\mu^2\phi+c\mu\phi+\mu^2w+\mu^2v+c\mu v+\mu^3\\
&+c\mu^2\ ,\\
b_4 =\;& -(P^*\mu p\alpha\beta\gamma\lambda)+P^*\mu\alpha\beta\gamma\lambda+P^*\mu p\beta\gamma\lambda+P^*cp\beta\gamma\lambda+\mu w\gamma\lambda+\mu v\gamma\lambda+cv\gamma\lambda+\mu^2\gamma\lambda+c\mu\gamma\lambda\\
&+P^{*2}\phi\alpha\beta^2\lambda+P^{*2}\mu\alpha\beta^2\lambda+P^*v\phi\alpha\beta\lambda+P^*\mu\phi\alpha\beta\lambda+P^*\mu v\alpha\beta\lambda+P^*\mu^2\alpha\beta\lambda+P^*w\phi\beta\lambda\\
&+P^*\mu\phi\beta\lambda+P^*c\phi\beta\lambda+P^*\mu w\beta\lambda+P^*\mu^2\beta\lambda+P^*c\mu\beta\lambda+\mu w\phi\lambda+\mu v\phi\lambda+cv\phi\lambda+\mu^2\phi\lambda+c\mu\phi\lambda\\
&+\mu^2w\lambda+\mu^2v\lambda+c\mu v\lambda+\mu^3\lambda+c\mu^2\lambda-A^*P^{*2}\mu\alpha'\beta^2\theta-A^*P^{*2}c\alpha'\beta^2\theta-P^*S^*\mu\alpha\beta^2\theta\\
&-A^*P^*\mu\alpha\beta^2\theta+A^*P^{*2}c'\alpha\beta^2\theta-A^*P^*c\alpha\beta^2\theta-A^*P^*\mu w\alpha'\beta\theta-A^*P^*\mu v\alpha'\beta\theta-A^*P^*cv\alpha'\beta\theta\\
&-A^*P^*\mu^2\alpha'\beta\theta-A^*P^*c\mu\alpha'\beta\theta-A^*\mu w\alpha\beta\theta-A^*\mu v\alpha\beta\theta+A^*P^*c'v\alpha\beta\theta-A^*cv\alpha\beta\theta-A^*\mu^2\alpha\beta\theta\\
&+A^*P^*c'\mu\alpha\beta\theta-A^*c\mu\alpha\beta\theta-S^*\mu w\beta\theta+A^*P^*c'w\beta\theta-S^*\mu v\beta\theta-S^*cv\beta\theta-S^*\mu^2\beta\theta-S^*c\mu\beta\theta\ ,
\end{aligned}
\tag{S18}
$$

and $\alpha = \alpha(P^*), c = c(P^*), \alpha' = \alpha'(P^*), c' = c'(P^*)$ for brevity.

Noting that b_1 is always positive, the endemic equilibria E^* has two purely imaginary eigenvalues (with the other two eigenvalues having non-zero real part) when (1) $b_3 > 0$, (2) $b_4 \neq 0$, and (3) $b_1b_2b_3 - b_1^2b_4 - b_3^2 = 0$ [Asada and Yoshida, 2003]. For a Hopf bifurcation to occur, we must also satisfy any non-degeneracy conditions: for instance, we also require that for the value $\beta = \beta^H$ that satisfies the above conditions, $\frac{d}{d\beta}[b_1b_2b_3 - b_1^2b_4 - b_3^2]|_{\beta=\beta^H} \neq 0$, meaning the complex eigenvalues actually cross the imaginary axis as β changes [Asada and Yoshida, 2003].

The condition for a Hopf bifurcation to occur at the secondary endemic equilibrium E_2^* is the same,

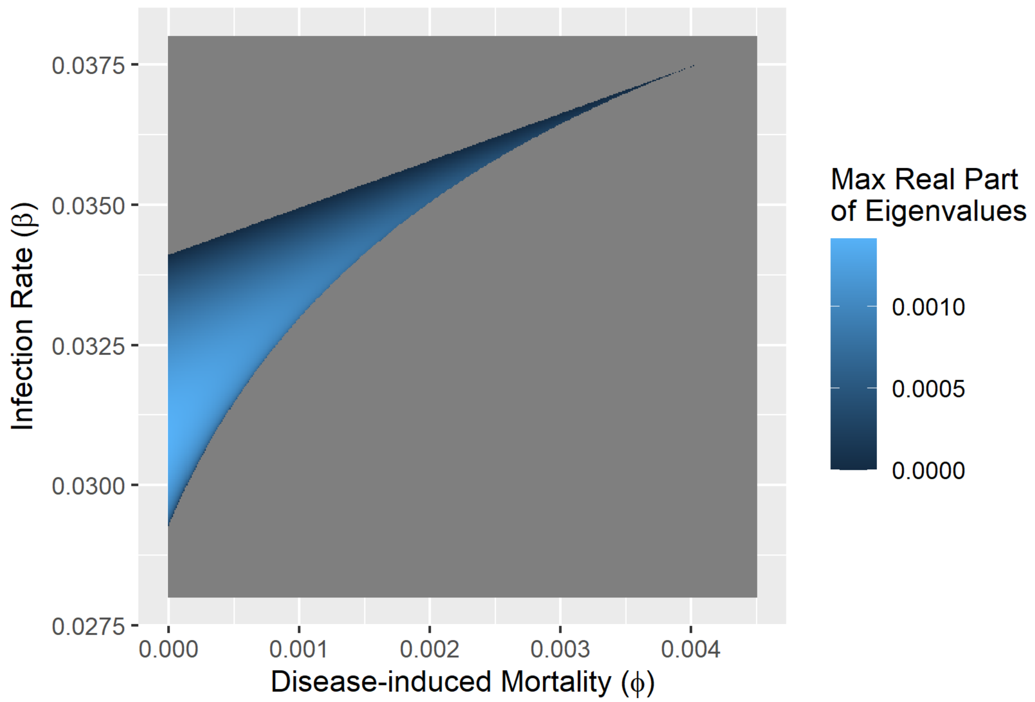

Figure S2: The largest real part of the four eigenvalues of the Jacobian evaluated at E_2^*, as the infection rate (β) and disease-induced mortality rate (ϕ) are varied. Over the entire area it exists (the non-grey region), E_2^* always has one eigenvalue with positive real part, meaning that it is always unstable. Other parameter values were the same as Figure 6.

but substituting S_2^*, A_2^*, P_2^* for S^*, A^*, P^*. However, numerical simulations support that there are no Hopf bifurcations around E_2^* for the parameters used in Figure 6. Figure S2 shows the largest real part of the four eigenvalues of the Jacobian at E_2^*. Over the entire area that E_2^* exists, it is always unstable. Since E_2^* never changes in stability, no Hopf bifurcations occur at E_2^*.

S1.7 Additional MatCont Figures

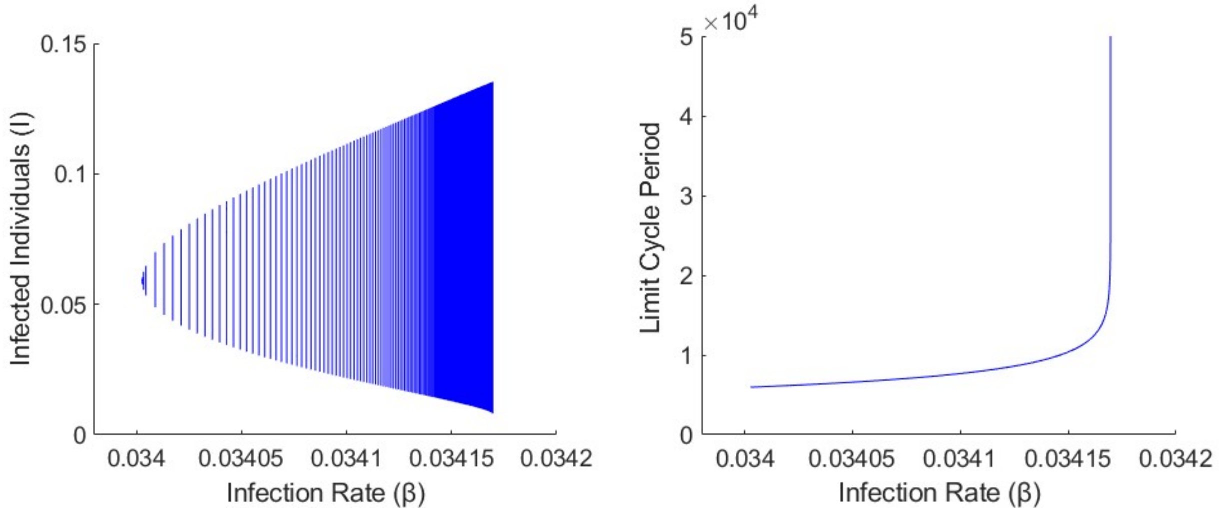

Figure S3: A demonstration that for $\phi < 0.001304$ (beneath the neutral saddle point), the homoclinic bifurcation in Figure 6 involves an unstable limit cycle. The left plot shows the size (min/max number of infected individuals) of the resulting limit cycle, while the right plot shows the period of the limit cycle. As the infection rate β increases, a subcritical Hopf bifurcation results in an unstable limit cycle, which is then destroyed by a homoclinic bifurcation. Parameters values were $\phi \approx 0.00115$, $\alpha(P) = \alpha_0 = 0.2$, $c(P) = c_0 = 0$, $\mu = 0.0006$, $\gamma = 0.04$, $\theta = 1$, $\lambda = .24$, $p = .3$, $w = 0$ and $v = 0.005$.

S1.8 Additional Simulation Results

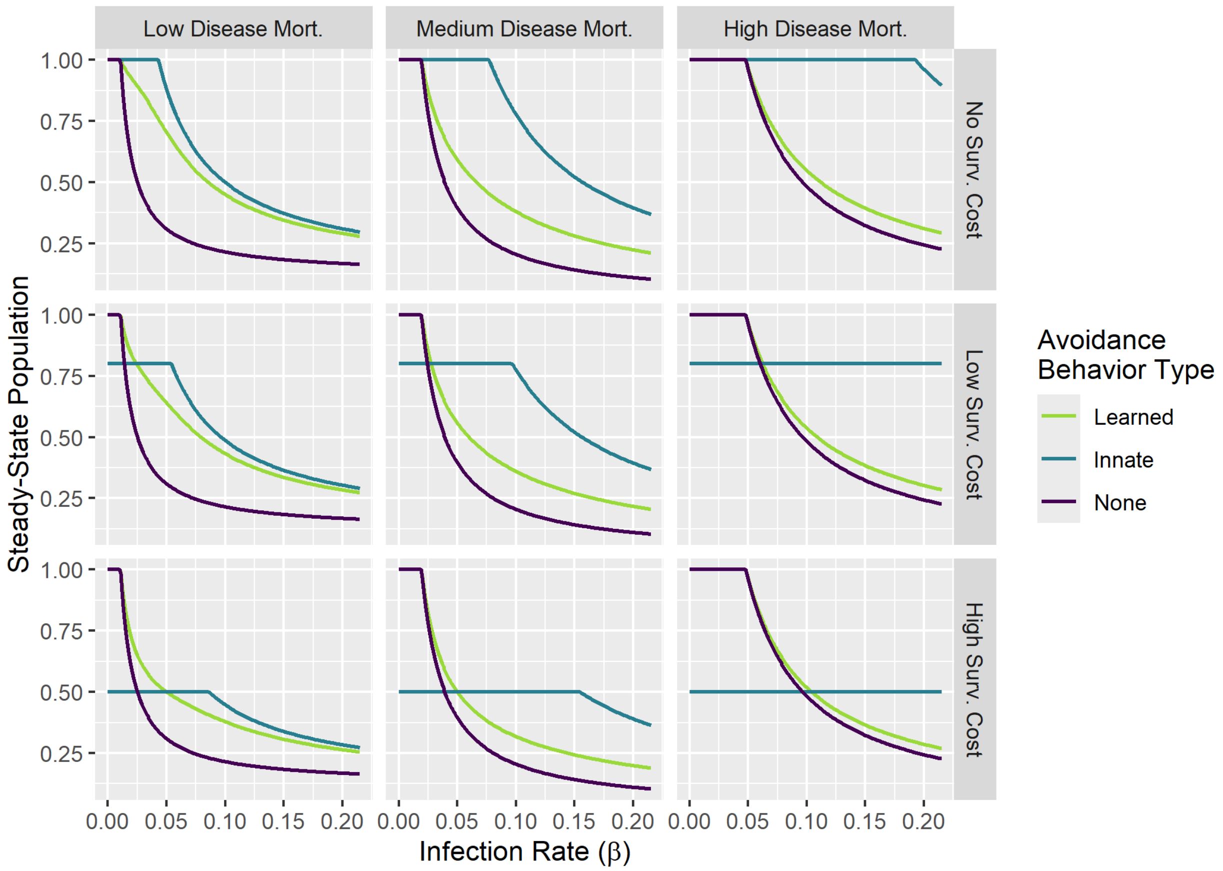

Figure S4: The steady-state population size resulting from learned, innate, or no behavioral avoidance as the infection rate (β) varies. With more effective behavioral avoidance ($\alpha(P) = \alpha_0 = 0.25$), greater steady-state populations are achieved by the innate and learned avoidance models than in Figure 9. Simulations assume constant avoidance behavior, with the survival cost $c(P) = c_0$ equal to 0 (no cost), 0.25μ (low cost), or μ (high cost). The no avoidance model was obtained by setting $\alpha_0 = 1$ and $c_0 = 0$. Disease-induced mortality rates were $\phi = 0.0044$ (low), 0.04 (medium), or 0.16 (high). Other parameter values were $\mu = 0.0006$, $\gamma = 0.04$, $\theta = 1$, $\lambda = 0.24$, and for the learned avoidance model, $p = 1, w = 0, v = 0$.

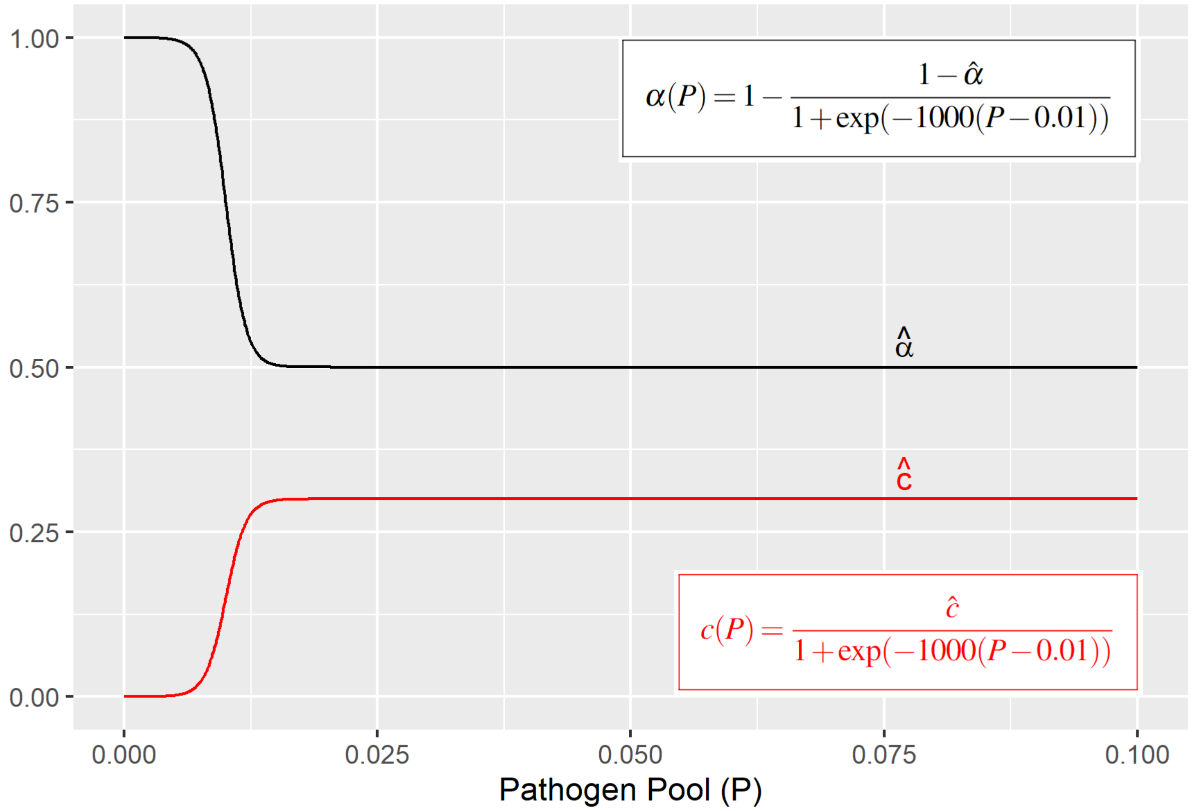

Figure S5: A simple example of non-constant avoidance behavior. There is a shift between two different modes of behavior around $P = 0.01$, represented in the functions $\alpha(P)$ (avoidance effectiveness, black) and $c(P)$ (survival cost of avoidance, red). Avoidance behavior may 'turn off' when P is small if the pathogen is no longer detected, or if the pathogen is not 'worth avoiding' at small concentrations.

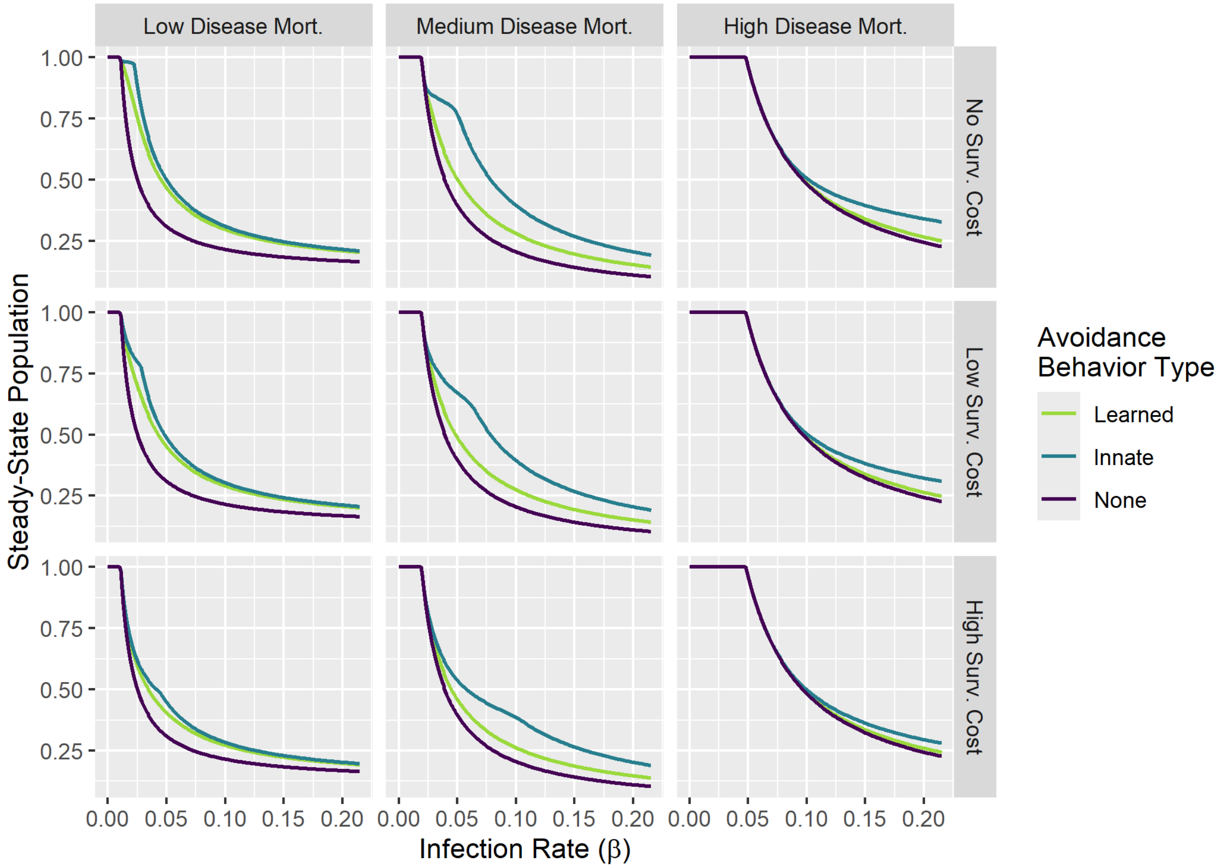

Figure S6: The steady-state population size resulting from learned, innate, or no behavior using the forms of the non-constant avoidance functions shown in Figure S5 (with $\hat{\alpha} = 0.5$ and $\hat{c} = 0, 0.25\mu, \mu$ for no, low, and high cost scenarios). Since there is no survival cost when the disease is not present, innate avoidance doesn't have the same downsides as in Figure 9. The no avoidance model was obtained by setting $\hat{\alpha} = 1$ and $\hat{c} = 0$. Disease-induced mortality rates were $\phi = 0.0044$ (low), 0.04 (medium), or 0.16 (high). Other parameter values were $\mu = 0.0006$, $\gamma = 0.04$, $\theta = 1$, $\lambda = 0.24$, and for the learned avoidance model, $p = 1, w = 0, v = 0$.

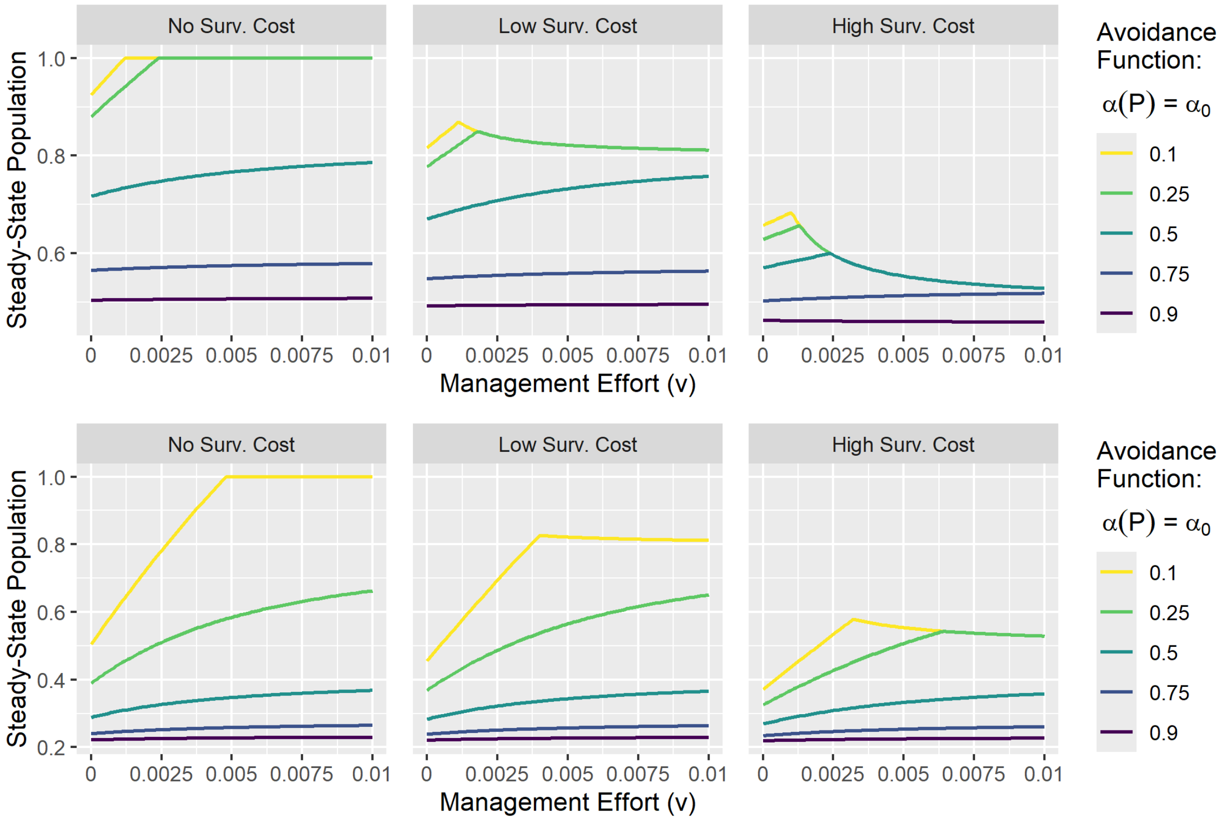

Figure S7: The steady-state population size resulting from varying levels of management effort to induce behavioral avoidance (v) in the learned avoidance system. **Top:** $\phi = 0.0044$, and $R_0 = 2.5$ at $v = 0$. **Bottom:** $\phi = 0.04$, and $R_0 = 5$ at $v = 0$. Compared to Figure 10, management became less effective under lower disease-induced mortality and a higher initial R_0. Simulations assume constant avoidance behavior, with the survival cost $c(P) = c_0$ equal to 0 (no cost), 0.25μ (low cost), or μ (high cost). Other parameter values were $\mu = 0.0006$, $\gamma = 0.04$, $\theta = 1$, $\lambda = 0.24$, $p = 1$, $w = 0$.
